# Supplementary material for: Trachoma prevention practice and associated factors among mothers having children aged under nine years in Andabet district, northwest Ethiopia, 2022: A multi-level analysis
Source: PLoS Negl Trop Dis. 2023 Jun 30;17(6):e0011433. doi: 10.1371/journal.pntd.0011433 (PMC10313038; doi:10.1371/journal.pntd.0011433)
Supplement: Attachment — Submitted filename: PNTD-D-22-01493-Koroma comments.pdf [file pntd.0011433.s002.pdf]

PLOS Neglected Tropical Diseases

Trachoma prevention practice and associated factors among mothers having children aged under nine years in Andabet district, northwest Ethiopia, 2022 :a multi-level analysis

--Manuscript Draft--

**Manuscript Number:**	PNTD-D-22-01493
**Full Title:**	Trachoma prevention practice and associated factors among mothers having children aged under nine years in Andabet district, northwest Ethiopia, 2022 :a multi-level analysis
**Short Title:**	Trachoma prevention practice and associated factors
**Article Type:**	Research Article
**Keywords:**	Key word: Trachoma, Trachoma prevention practice, multi-level analysis, random effect analysis.
**Abstract:**	AbstractBackgroundThe world health organization adopted the Surgery, Antibiotic, facial cleanness and environmental improvement (SAFE) strategy for prevention of trachoma and different prevention strategies were implemented in Andabet district. However, the prevalence of trachoma is still higher so it is very important to assess the ground trachoma prevention practice since there is inadequate study on the study area. This study was aimed to assess the magnitude of trachoma prevention practice and the effect of different factors on trachoma prevention practice at different community level.ObjectiveThe study was aimed to assess trachoma prevention practice and associated factors among mothers having children under nine years in Andabet district,Northwest Ethiopia.MethodA community-based cross-sectional study involving 624 participants was conducted from June 1- 30, 2022. Systematic random sampling was conducted to select study participants. Multi-level binary logistic regression analysis was used to identify factors associated with poor TPP. Descriptive and summary statistics were performed and Variables with p value < 0.5 in the best fitted model were declared to be significantly associated with poor TPP.ResultsIn this study, proportion of poor TPP was found to be 50.16% (95%CI=46.23, 54.08). In the multi variable multi-level logistic regression; having no formal education (AOR= 2.95; 95%CI: 1.41,6.15) and primary education (AOR= 2.33; 95%CI:1.04, 5.24), being farmer (AOR=3.02; 95%CI:1.73,5.28), and merchant (AOR=2.63; 95%CI:1.20, 5.75), time taken to water point >30 minutes (AOR=4.60,95CI:1.30,16.26) and didn't take health education about trachoma (AOR=2.36;95CI:1.16,4.79) were significantly associated with poor TPP.<br>Conclusion and recommendationThe proportion of poor trachoma prevention practice was high relative to other studies. Level of education, occupation, time taken to water source and health education were significantly associated with poor trachoma prevention practice.
**Additional Information:**	
**Question**	**Response**
**Government Employee**<br><br>Are you or any of the contributing authors an employee of the United States government?<br><br>Manuscripts authored by one or more US Government employees are not copyrighted, but are licensed under a CC0 Public Domain Dedication, which allows	No - No authors are employees of the U.S. government.

Powered by Editorial Manager® and ProduXion Manager® from Aries Systems Corporation

unlimited distribution and reuse of the article for any lawful purpose. This is a legal requirement for US Government employees.<br><br>This will be typeset if the manuscript is accepted for publication.	
**Financial Disclosure**<br><br>Enter a financial disclosure statement that describes the sources of funding for the work included in this submission and the role the funder(s) played. This includes grants and any commercial funding of the work or authors.<br><br>This statement will be typeset if the manuscript is accepted for publication.<br><br>Review the submission guidelines and the instructions link below for detailed requirements and guidance.	The University of Gondar funded this study. The funding institution did not involve in the design of the study, data collection, analysis, or interpretation of the results and manuscript writing.
**Competing Interests**<br><br>On behalf of all authors, disclose any competing interests that could be perceived to bias this work.<br><br>This statement will be typeset if the manuscript is accepted for publication.<br><br>Review the instructions link below and PLOS NTDs' competing interests policy to determine what information must be disclosed at submission.	The authors declare that they have no competing interest
**Data Availability**<br><br>Provide a **Data Availability Statement** in the box below. This statement should detail where the data used in this submission can be accessed. This statement will be typeset if the manuscript is accepted for publication.	The data set used and analyzed during this study is available at UOG

Before publication, authors are required to make all data underlying their findings fully available, without restriction. Review our PLOS Data Policy page for detailed information on this policy. Instructions for writing your Data Availability statement can be accessed via the Instructions link below.	

Manuscript
Click here to access/download;Manuscript;manuscript TPP.docx

Trachoma prevention practice and associated factors among mothers having children aged under nine years in Andabet district, northwest Ethiopia, 2022 :a multi-level analysis

Zufan Alamrie Asmare [1*], Natnael Lakachew Assefa [2], Dagmawi Abebe[3], Solomon Gedlu Nigatu [4], Yezinash Addis Alimaw [2]

1 Department of ophthalmology, School of Medicine and Health Science, Debre Tabor University, Debre Tabor, Ethiopia

2 Department of optometry, School of Medicine and Health Science, University of Gondar, Gondar, Ethiopia

3 Department of ophthalmology, School of Medicine and Health Science, University of Gondar, Gondar, Ethiopia

4 Department of Epidemology & biostatistics, School of Medicine and Health Science, University of Gondar, Gondar, Ethiopia

***corresponding author**

Zufan Alamrie Asmare (Debre Tabor University School of Medicine and Health Science)

EMAIL: zufanalamrie2@gmail.com

PHONE NO; +251937374268,

P.O BOX- 272

Abstract

Background

The world health organization adopted the Surgery, Antibiotic, facial cleanness and environmental improvement (SAFE) strategy for prevention of trachoma and different prevention strategies were implemented in Andabet district. However, the prevalence of trachoma is still higher so it is very important to assess the ground trachoma prevention practice since there is inadequate study on the study area. This study was aimed to assess the magnitude of trachoma prevention practice and the effect of different factors on trachoma prevention practice at different community level.

community levels (plural)

Objective

Objective almost same as aim

The study was aimed to assess trachoma prevention practice and associated factors among mothers having children under nine years in Andabet district,Northwest Ethiopia.

Method

A community-based cross-sectional study involving 624 participants was conducted from June 1- 30, 2022. Systematic random sampling was conducted to select study participants. Multi-level binary logistic regression analysis was used to identify factors associated with poor TPP. Descriptive and summary statistics were performed and Variables with p value < 0.5 in the best fitted model were declared to be significantly associated with poor TPP.

Results

In this study, proportion of poor TPP was found to be 50.16% (95%CI=46.23, 54.08). In the multi variable multi-level logistic regression; having no formal education (AOR= 2.95; 95%CI: 1.41,6.15) and primary education (AOR= 2.33; 95%CI:1.04, 5.24), being

farmer (AOR=3.02; 95%CI:1.73,5.28), and merchant (AOR=2.63; 95%CI:1.20, 5.75), time taken to water point >30 minutes (AOR=4.60,95CI:1.30,16.26) and didn’t take health education about trachoma (AOR=2.36;95CI:1.16,4.79) were significantly associated with poor TPP.

Conclusion and recommendation

There is no recommendation. Consider removing 'and recommendation' if you want to keep word count low.

The proportion of poor trachoma prevention practice was high relative to other studies. Level of education, occupation, time taken to water source and health education were significantly associated with poor trachoma prevention practice.

Key word: Trachoma, Trachoma prevention practice, multi-level analysis, random effect analysis.

Author summary

a neglected tropical disease; no 's'.

Trachoma is a neglected tropical diseases, It has been one of the most devastating diseases affecting 60 to 90 % of children, especially under nine years. In Ethiopia there are 10.2 million cases of trachoma, in which Amhara region takes the lion share. Trachoma elimination has focused on the Surgery & Antibiotic elements of the SAFE strategy. However, Facial cleanliness & Environmental improvements are critical for sustained progress towards elimination. The rationale behind F and E components of SAFE strategy is that changes in hygiene behavior and improvement in environmental infrastructure might be able to reduce prevalence of trachoma and thus be an ideal long term strategies for trachoma control. This paper adds to the literature by illustrating the status of trachoma prevention practice strategy and associated factors in Ethiopia and may support trachoma elimination in Ethiopia and globally.

lion's share

no 'an'

Consider rewriting this section: Half global population requiring intervention for trachoma elimination is in Ethiopia where some regions/districts have up to 37% TF rate after years of A treatment. This study demonstrates the need to consider support for the introduction of other interventions (F and E) for trachoma elimination in Ethiopia and thus elimination of an estimated half the global burden.

Introduction

Trachoma is caused by *Chlamydia trachomatis*, a neglected tropical diseases (1, 2). It has been one of the most devastating diseases affecting 60 to 90 % of children, especially under nine years (3, 4).Trachoma affects their school performance, inability to lead a healthy and productive life (3). Moreover, this devastating diseases can result in vision loss, stigma, reduced productivity and economic loss of US$ 2.9-5.3 billion annually, and half of the burden of trachoma is shared by Ethiopia solely (5, 6).

Globally, trachoma causes 1.9 million visual impairments and 1.2 million blindness. It is still endemic in 44 countries (2, 7). More than 80% of active trachoma is concentrated in Africa, nearly half is in sub-Saharan Africa (8). In Ethiopia there are 10.2 million cases of trachoma, in which Amhara region takes the lion share (62.6%) (5, 9).

According to different studies conducted in Ethiopia, the prevalence of poor prevention practice is high, which ranges between 45.5 and 64.4% (1, 10). Evidences have shown that individual level factors such as age of the mother, husband education, basic knowledge about trachoma, mothers attitude towards trachoma, taking health education about trachoma, time taken to water point and frequency of getting water and also community level factors such as Residence and types of water source were affect trachoma prevention practice (1, 10-19)

Even though there are different strategies to tackle trachoma, like WHO which implemented SAFE (surgery for advanced disease, Antibiotic, facial cleanliness and Environmental improvement) to control the diseases, working on prevention practice has a paramount advantage since poor prevention practice is primarily responsible for the increasing prevalence of trachoma (11, 20-22).

Proper practicing of F (facial cleanness) and E (Environmental improvement) is responsible for 58.7% reduction of trachoma prevalence at all ages and 37.4% in

children (23). moreover, trachoma was disappeared from most of United States of America and Western Europe before antibiotics was discovered (23)

The rationale behind F and E components of SAFE strategy is that changes in hygiene behavior and improvement in environmental infrastructure might be able to reduce prevalence of trachoma and thus be an ideal long term strategies for trachoma control (24).

Good argument for introduction of F and E especially with the results of

After 8 to 11 years implementation of SAFE in the study area Andabet, North west Ethiopia , the prevalence of TF (trachomatous follicular) was 37% in 2017. Which is hyper endemic; other than the other 3 similar settings (9, 15). Studies on the scope of trachoma prevention practice (F and E components of SAFE) and its associated factors are limited. Even those few studies are failed to account for the existence of community level variations of trachoma prevention practice. In order to have good trachoma prevention practice, interventions at both individual and community level factors should be taken in to account.

Therefore, this study aimed to assess trachoma prevention practice and associated factors among mothers having children aged under nine years in Andabet district with a focus on both individual and community level factors. Finally, the findings of this study will be used as a base line for responsible bodies (policy makers, governmental and non-governmental organizations) in order to have appropriate decision and for further investigation by future researchers.

Methods

Study area and period

This study was conducted in Andabet district, south Gondar zone, Amhara region, Ethiopia from June 1-30, 2022.The district is located 717 km from Addis Ababa and

150km east of Bahirdar, the capital city of Amhara region. Its total population is 152,683 and according to 2022 data of Andabet district administration office; the district has 26 kebeles with 34765 household. It has 1 primary health care center and 2 health posts. Its climate condition is Woinadega

Study design

Community based cross-sectional design was used with systematic random sampling method from June 1-30, 2022.

Source and study population

The source and study population of the study were Mothers having children aged under nine years who had been living in Andabet district, Northwest Ethiopia. Mothers who had at least one child of age less than nine years and those lived in the district at least for six months were included in the study.

Exclusion criteria

Mothers with mental illness, other serious systemic illness, and hearing problem were excluded from the study.

Is the proportion of women excluded significant. Please provide rationale for the exclusion

Why? Is this not discrimination? what about sign languages for hearing problems, other systemic illness?

Sample size determination

Sample size for the proportion of trachoma prevention practice

The sample size (n) was determined using single population proportion formula by taking Proportion of poor practice is 48.5% from similar study in Oromia

Please consider finding someone who can proofread and edit to

My statistics is limited to assess sample size selection

$n= (Z\alpha/2)^2 P\ (1-P))/d2$

n – Sample size

Z - Confidence interval at 95%= 1.96

P – Proportion of poor practice is 48.5% from similar study in Oromia, Ethiopia (12).

d - Marginal error=5%

$n=1.96^{2} \times .485 \times .515 / (0.05 \times 0.05) = 384$

Finally, after considering a 10% non- response rate and design effect (1.5), the required sample size for this particular study was 634.

Ethical consideration

The study adhered to the tenets of the Declaration of Helsinki and approval was sought and obtained from the Ethical Review Board of College of Medicine and Health Sciences, University of Gondar. Permission letter was obtained from Andabet district administrative office and written informed consents were obtained from all voluntary participants. The participants were informed of the study would not impose harm to them. There were no personal identifiers and confidentiality of the study participants were maintained at all stages of data processing.. Informed verbal consent was obtained from each respondent and Confidentiality was kept by using codes and by avoiding personal identifiers.

Sampling technique and procedure

A multistage sampling technique was used during the sampling process. Six Kebeles out of 26 Kebeles was selected by using a simple random sampling method after a list of kebeles was obtained from the Andabet district administration bureau. The total sample size of the study was allocated proportionally for each Kebele based on number of mothers having children aged under nine years that were found in each Kebele. Finally, the households were chosen using a systematic random sample technique.

To carry out systematic random sampling, sampling frames were collected from each kebele. The total estimated number of study population was 4565. Based on study population and sample size required from each kebele, we calculated the interval between households and found to be seven. Then the first household was randomly selected from 1 to 7 serial number of sampling and the remaining households were selected at every 7th interval. If mothers in the household did not fulfill the inclusion criteria and/or did not found with two repeated visits the next household was taken.

Variables of the study

Dependent variables

The dependent variable was mother's trachoma prevention practice.

Independent variables

For this study the independent variables were classified as individual and community level variables. The individual level variables were Age of the mother, Mother education ,Husband education ,Occupation of the mother ,Religion, Marital status Number of children under nine ,age of the youngest child, Sex of the youngest child, Basic trachoma Knowledge, Attitude towards trachoma and Amount of water used per person per day. Community level variables for this study were Residence,Type of water source and Community women illiteracy level.

Operational definition

Trachoma prevention practice: It is the assessment of mother practice towards two out of four components of WHO SAFE strategy and is only facial cleanness (F) and environmental hygiene (E).it was Measured using 10 items all yes/no and classified as good and poor based on the mean value (1).

Knowledge: of mother's knowledge towards trachoma was assessed, using 15 yes/no questions and classified as good and poor based on the mean of the scores (10)

Attitude: assessed using 7 agree/disagree statements and classified as good and poor based on mean of score (10). Those who scoring greater than the mean were considered to have good attitude (10, 25, 26)

Facial cleanliness: measured as absence of ocular discharge, nasal discharge, and fly (s) on eye during the time of examination. If there is one from the listed, it is considered as not clean (27).

Ocular discharge: Is any discharge around and/or in the eye at time of examination (27).

Nasal discharge: any discharge seen in the nose at time of examination (27).

Fly-eye: at least one fly contact with the eyelid margin during eye observation (18).

Time taken to water source: collection time does not exceed 30 minutes.

Availability of water: An average person uses about 20 liters of water per day for Domestic and personal hygiene (28).

Utilization of waste disposal pit: if there is discarded unwanted agricultural product in the pit , if there is domestic products in the pits or if there is presence of ashes (burned sign of waste) ,it was considered as utilized, otherwise not (29).

Latrine utilization: Latrines that displayed at least two signs of latrine utilization during the observation were considered latrine utilization "signs of use," such as footpath to the Latrine, fresh excreta inside the latrine, and presence of splash of urine, and absence of spider web of the squat. If there were at least two from the above it was considered as utilized (30).

Cleanness of compound: if the household (residential) compound was free from solid waste, liquid wastes, feces, animal dungs, and domestic waste, it was considered as clean (10).

Health education: was acquired education about trachoma and trachoma prevention practice at least once in the previous two years (29).

Cleanness of latrine: if there is at least one from these; human excreta out of the pit, stagnant urine, and unwanted trash in the floor of latrine, it was not considered as clean (31) .

Community women illiteracy: it is the aggregated community level variable derived from maternal educational level and measured as the proportion of women with no formal education at the kebele/community level. Based on a median value it was then dived into low (mothers from communities with lower illiteracy level) and high (mothers from communities with higher illiteracy level) categories (32, 33)

Data collection procedure and quality control

Quantitative data was collected through face to face interview and observation. Which were adapted from different literatures and modified to the context. Data was collected by trained four BSc ophthalmic nurses using interviewer administered Amharic version questioner which is translated from English language and through observation.

One day training was given to data collectors by the principal investigator. The principal investigator supervised the data collection on daily bases. Pre-testing was conducted on 5% of participants that was from Kebele, which was not selected for the study.

Based on the finding of the pre-test, data collectors were re-oriented and the questionnaire was modified accordingly. The reliability of the question for trachoma

prevention practice was checked by crombach alpha and the scale reliability coefficient was 0.795

Data processing and Statistical analysis

Data was entered into Epi-Data version 4.6 then data cleaning and coding was done using STATA version 16. STATA was used for analyses (both descriptive and analytical analyses). Descriptive statistics were reported using text, tables, and figures. Proportion of poor trachoma prevention practice with its 95% Confidence interval (CI) was reported. To assess factors associated with trachoma prevention practice, a multilevel logistic regression analysis was used. This is used because of mothers are nested in kebeles/community, which violet the independent assumption of the standard logistic regression model. Besides, the random effect parameters and the sampling technique also dictate us to use a multilevel analysis.

While conducting a multilevel binary logistic regression analysis, we fitted both random effect and fixed effect analyses. The random effect parameter, intraclass correlation coefficient (ICC) quantifies the degree of heterogeneity of trachoma prevention practice between clusters and ICC of more than 10% indicates that accounting the cluster level variability of trachoma preventive practice using multi -level analysis is appropriate. Moreover, proportion change in variance (PCV), and median odds ratio (MOR) were assessed.

In fixed effect analysis, four models were fitted; model 1 (with the outcome variable only), model 2 (incorporating individual level variables), model 3 (fitted with community level variables), and model 4 (incorporating both individual and community level variables simultaneously). Among the four models fitted, the last model (model 4) was selected as the best fitted model since it has the lowest deviance and highest PCV.

Eligible variables for the multivariable multilevel analysis were selected if their p value in the bivariable analysis was <0.25. For all models fitted, the adjusted odds ratio (AOR) with its 95% CI were reported. However, the interpretations are based on the final model, the best fit model. Finally, variables with p<0.05 in the final model were declared to be significantly associated with trachoma prevention practice.

Result

Socio-demographic characteristics of study participants

Four hundred and ninety six

A total of 624 study participants were included in the study.Forty hundred ninety-six (79.49%), mothers had children aged less than or equal to two years with a mean age of 2. Three hundred fifty-seven (57.2%) mothers did not receive formal education and 554 (88.78%) mothers didn't got health education about trachoma (Table 1).

Environmental and related characteristics

Around three-fourth (73.24%) of mothers got water from river and 342 (54.81%) of them travel more than 30 minutes to get water. Around two third (64.9%) and 354(56.73%) of mothers had poor basic trachoma knowledge and attitude towards trachoma. Regarding cleanness of the compound, 448 (71.79%) of mothers had unclean house compound (Table 2)

Magnitude of Trachoma prevention practice

The overall proportion of poor trachoma prevention practice was 50.16 (95%CI: 46.23%, 54.08%) (Figure 1). More than two third of mothers had clean face, 427 (68.43%) and three fourth of children in the study had unclean face, 457 (73.24%). More than three fourth (77.40%) of the mothers uses latrine (Table 3).

Factors associated with trachoma prevention practice

In the multivariable multi-level logistic regression, mothers who didn't take formal education and who had primary education level had 2.95 and 2.33 times higher odds of poor trachoma prevention practice as compared to those mothers who had secondary and above education level (AOR=2.95; 95%CI:1.41,6.15) and (AOR= 2.33; 1.04,5.24) respectively.

Mothers who were farmer and merchant had 3.02 and 2.63 times higher odds of poor trachoma prevention practice as compared to those mothers who were housewife (AOR=3.02; 95%CI:1.73, 5.28) and (AOR=2.63; 95%CI:1.20, 5.75) respectively.

Regarding time taken to water point, mothers who traveled >30 minute to the water point had 4.60 (AOR= 4.60, 95CI:1.30, 16.26) times higher odds of poor trachoma prevention practice as compared to those mothers who traveled ≤30 minute. Mothers who didn't take health education about trachoma had 2.36 (AOR= 2.36; 95%CI:1.16, 4.79) times higher odds of poor trachoma prevention practice as compared to those mothers who had taken health education about trachoma (Table 5)

Discussion

The aim of this study was to assess trachoma prevention practice and associated factors in andabet district, Northwest Ethiopia. In this study, the proportion of poor trachoma prevention practice was 50.16%. This finding is in line with a study conducted in Oromia Ethiopia (1). However, it is lower than a study conducted in Tigray.(10).The possible explanation for this discrepancy with a study done in tigray might be community based trachoma interventions, particularly those focused on increasing awareness about the diseases have periodically expanded and organizations like carter have been doing their duties in our study area (34). Our study finding is higher than a study conducted in north

Vietnam and lemo district of southern Ethiopia (12, 35) The discrepancy from study done in vetnam might be due to sociocultural and economic difference that is Vietnam is relatively developed country with accessible water and health service like well oriented health education programs about trachoma (36) and also the study participants of a study done in Vietnam are school aged children who got trachoma prevention related information from their school (35).while the discrepancy in lemo might be due to study time period difference.

The study at hand found that maternal educational level is associated with trachoma prevention practice. Mother who didn't take formal education and had primary education level had higher odds of poor prevention practice as compared with those mothers who had secondary and above education level. The possible explanation may be those who didn't take formal education and only receive primary education lose a thorough understanding of the disease, mechanism of transmission and prevention mechanisms; therefore, they might not take measures to prevent trachoma and the odds of poor practice might be increased (12) (35, 36)

Having not taken health education about trachoma is also associated with higher odds of poor trachoma prevention practice as compared to their counterparts in the current study. This finding is supported by study conducted in lemo district (12). This is due to the fact that mothers who didn't take health education program are more vulnerable to cultural misconceptions about use water for environmental sanitation and personal hygiene (11, 37) Besides, mothers who didn't got health education program didn't have practice skills needed to adopt for prevention of trachoma (11) (37, 38)

Maternal occupation is another factor that is associated with trachoma prevention practice in which mothers whose occupation is farmer and merchant is associated with

higher odds of poor trachoma prevention practice as compared to being housewife. The possible reason might be according to our study 88% of farmers and 93.6% of merchants didn't take health education about trachoma. Also, 81.5% of farmers travel more than 30 minutes to get water. The other possible reason might be those mothers who are farmer and merchant are less efficient in terms of time for facial cleanness and environmental improvement than housewives.

Consistent with other studies conducted in Kenya and Oromia (1, 39), in this study, longer time to water point is significantly associated with poor trachoma prevention practice; those who took greater than 30 minutes to water point was associated with higher odds of poor trachoma prevention practice as compared to those who takes less than or equal 30 minutes to the water point. This may be due to the link between time wasted getting water and water scarcity. The more loss of time to get water, the more they save the water and the shortage of water in turn deteriorates the hygienic practice of mothers (39).

7 Strength and limitation of the study

This study assessed important topics that has both clinical and public health importance accounting the variability of poor trachoma prevention practice between kebeles/communities. The use of multi-level logistic regression analysis allows to have `the appropriate estimate. However, this study is not without limitation. Social desirability bias might be introduced while assessing sensitive variables such as (fomite sharing practice and infant feces disposal to latrine). In addition it lucks repeated observation and this may alter the outcome variable in either direction

8 Conclusion

The proportion of poor trachoma prevention practice was high relative to other studies. Those who didn't take formal education, who had primary education level, those who take more time to water point (>30 minutes), those who didn't taken health education about trachoma and those who were farmer and merchant were at higher odds of poor trachoma prevention practice.

9 Recommendations

To governmental and non-governmental organizations: focus on facial cleanness (F) and Environmental improvement (E) components of SAFE.

To amhara region trachoma control program: planning health education program and enhancing water supply are recommended to improve trachoma prevention practice overall.

Any recommendation for the global trachoma community who should be very interested in what ha[pens in Ethiopia?

To Andabet district health office: special attention should be given for those mothers with no formal education and primary education and who are away from water source. Besides, health education programs about trachoma should be strengthened.

Acknowledgment

First and foremost our special appreciation goes to university of Gondar for its financial support as well as giving this opportunity. We are also also delighted to thank the department of optometry and its staffs who support us while conducting this paper. Lastly but not least, we would like to give our appreciation and thanks to our study participants and data collectors.

References

1. Abera T, Tilahun W, Waqjira I. Trachoma prevention practice among mothers with child age of under-9 years and factors associated in rural district of Oromia Region, Ethiopia: Community based cross-sectional study. World Journal of Advanced Research and Reviews. 2021;10(1):245-57.

2. WHO. Trachoma. 7,march 2022.

3. Frick KD, Hanson CL, Jacobson GA. Global burden of trachoma and economics of the disease. The American journal of tropical medicine and hygiene. 2003;69(5_suppl_1):1-10.

4. ETAT. Trachoma - a women and children's health issue. 2020.

5. Stewart AE, Zerihun M, Gessese D, Melak B, Sata E, Nute AW, et al. Progress to eliminate trachoma as a public health problem in Amhara National Regional State, Ethiopia: results of 152 population-based surveys. The American journal of tropical medicine and hygiene. 2019;101(6):1286.

6. Bamani S, King JD, Dembele M, Coulibaly F, Sankara D, Kamissoko Y, et al. Where do we go from here? Prevalence of trachoma three years after stopping mass distribution of antibiotics in the regions of Kayes and Koulikoro, Mali. PLoS neglected tropical diseases. 2010;4(7):e734.

7. WHO. Weekly epidemological record. 2019.

8. Stewart AE, Zerihun M, Gessese D, Melak B, Sata E, Nute AW, et al. Progress to eliminate trachoma as a public health problem in Amhara National Regional State, Ethiopia: results of 152 population-based surveys. 2019;101(6):1286.

9. Nash SD, Astale T, Nute AW, Bethea D, Chernet A, Sata E, et al. Population-based prevalence of Chlamydia trachomatis infection and antibodies in four districts with varying levels of trachoma endemicity in Amhara, Ethiopia. The American journal of tropical medicine and hygiene. 2021;104(1):207.

10. Gebretnsae H, Mamo N, Teklemariam T, Fenta K, Gebrehiwet T, Berhe A, et al. Knowledge, attitudes, and practices about trachoma in rural communities of Tigray Region, Northern Ethiopia: implications for prevention and control. Journal of environmental and public health. 2020;2020.

11. Njomo DW, Karimurio J, Odhiambo GO, Mukuria M, Wanyama EB, Rono HK, et al. Knowledge, practices and perceptions of trachoma and its control among communities of Narok County, Kenya. Tropical diseases, travel medicine and vaccines. 2016;2(1):1-10.

12. MuluneshG M MS, Minichil B. Trahoma prevention practice and associated factors in rural lemo district. 2021.

13. Tadesse B, Worku A, Kumie A, Yimer SA. The burden of and risk factors for active trachoma in the North and South Wollo Zones of Amhara Region, Ethiopia: a cross-sectional study. Infectious diseases of poverty. 2017;6(1):1-12.

14. Asfaw M, Zolfo M, Negussu N, Tadesse F, Tadele T, Sisay A, et al. Towards the trachoma elimination target in the Southern region of Ethiopia: How well is the SAFE strategy being implemented? The Journal of Infection in Developing Countries. 2020;14(06.1):3S-9S.

15. Lavett DK, Lansingh VC, Carter MJ, Eckert KA, Silva JC. Will the SAFE strategy be sufficient to eliminate trachoma by 2020? Puzzlements and possible solutions. The Scientific World Journal. 2013;2013.

16. Aiemjoy K, Stoller NE, Gebresillasie S, Shiferaw A, Tadesse Z, Sewnet T, et al. 'If an eye is washed properly, it means it would see clearly': a mixed methods study of face washing knowledge, attitudes, and behaviors in rural Ethiopia. PLoS neglected tropical diseases. 2016;10(10):e0005099.

17. West SK, Nanji AA, Mkocha H, Munoz B, Gaydos C, Quinn TC. Evidence for contamination with C. trachomatis in the household environment of children with active Trachoma: A cross-sectional study in Kongwa, Tanzania. PLoS neglected tropical diseases. 2019;13(12):e0007834.

18. Golovaty I, Jones L, Gelaye B, Tilahun M, Belete H, Kumie A, et al. Access to water source, latrine facilities and other risk factors of active trachoma in Ankober, Ethiopia. PLoS One. 2009;4(8):e6702.

19. Musonda C. Association between water, sanitation and hygiene (wash) and prevalence of trachoma in Monze district of southern province, Zambia: The University of Zambia; 2020.

20. Aragie S, Wittberg DM, Tadesse W, Dagnew A, Hailu D, Chernet A, et al. Water, sanitation, and hygiene for control of trachoma in Ethiopia (WUHA): a two-arm, parallel-group, cluster-randomised trial. The Lancet Global Health. 2022;10(1):e87-e95.

21. Organization WH. Water sanitation & hygiene for accelerating and sustaining progress on neglected tropical diseases: a global strategy 2015-2020. World Health Organization, 2015.

22. Chikwanda M, Munyinda N, Mwale C, Mbanefo P, Banda TC, Mubita P. An association between water, sanitation, and hygiene (WASH) and prevalence of trachoma in Monze district of Southern Province, Zambia. Journal of Water, Sanitation and Hygiene for Development. 2021;11(3):453-60.

23. Khandekar R, Thanah TTK, Thi PD. Impact of face washing and environmental improvement on reduction of active trachoma in Vietnam—a public health intervention study. Ophthalmic Epidemiology. 2006;13(1):43-52.

24. Ejere HO, Alhassan MB, Rabiu M. Face washing promotion for preventing active trachoma. Cochrane Database of Systematic Reviews. 2015(2).

25. Hassali MA, Shafie AA, Saleem F, Farooqui M, Aljadhey H. A cross sectional assessment of knowledge, attitude and practice towards Hepatitis B among healthy population of Quetta, Pakistan. BMC public health. 2012;12(1):1-8.

26. AlRasheed MM, Alsugair AM, Almarzouqi HF, Alonazi GK, Aleanizy FS, Alqahtani FY, et al. Assessment of knowledge, attitude, and practice of security and safety workers toward the COVID-19 pandemic: a cross-sectional Study. Frontiers in public health. 2021:254.

27. Stocks ME, Ogden S, Haddad D, Addiss DG, McGuire C, Freeman MC. Effect of water, sanitation, and hygiene on the prevention of trachoma: a systematic review and meta-analysis. PLoS Med. 2014;11(2):e1001605.

28. Tadesse B, Worku A, Kumie A, Yimer SA. Effect of water, sanitation and hygiene interventions on active trachoma in North and South Wollo zones of Amhara Region, Ethiopia: A Quasi-experimental study. PLoS neglected tropical diseases. 2017;11(11):e0006080.

29. MuluneshG M MS, Minichil B. Trahoma prevention practice and associated factors in rural lemo district. 2021.

30. D A. Latrine Utilization and Associated Factors Among Rural Households in Dega Damot Woreda, West Gojjam Zone, Amhara Region, Ethiopia 2021.

31. T. MK. National guideline for water,sanitation and hygine for tanzania school. july,2016.

32. Teshale AB, Tesema GA, Tessema ZT. Spatial variations and associated factors of knowledge of ORS packet or pre-packaged liquids for the management of diarrhea among women of reproductive age in Ethiopia: A spatial and multilevel analysis. PloS one. 2021;16(3):e0247772.

33. Shah AR, Subramanyam MA. Community-level women's education and undernutrition among Indian adolescents: A multilevel analysis of a national survey. PloS one. 2021;16(5):e0251427.

34. Organization WH. WHO Alliance for the Global Elimination of Blinding Trachoma by the year 2020: Progress report on elimination of trachoma, 2013. Weekly Epidemiological Record= Relevé épidémiologique hebdomadaire. 2014;89(39):421-8.

35. Khandekar R, Bullard M, Thanh TTK, Binh TQ. Knowledge and practice related to trachoma among children in Vietnam: A cross-sectional study. Journal of health communication. 2004;9(1):77-83.

36. david.K. Water Supply and Sanitation in Vietnam. 2 JMP-UNICEF special tabulation per wealth quintile, using MICS 2013.
37. Kuper H, Solomon AW, Buchan J, Zondervan M, Foster A, Mabey D. A critical review of the SAFE strategy for the prevention of blinding trachoma. The Lancet Infectious diseases. 2003;3(6):372-81.
38. Asaolu SO, Ofoezie IE. The role of health education and sanitation in the control of helminth infections. Acta tropica. 2003;86(2-3):283-94.
39. PN. M. . Knowledge, Practices and Perceptionss of Trachoma and its' Influence (Association With) on Health Seeking Behaviour of Patients in Kajiado Central Sub County, Kajiado County, Kenya: COHES, JKUAT; . 2017.

Tables

Table 1:-socio demographic characteristics of mothers having children aged under nine years in Andabet, north west Ethiopia, 2022 :a multi-level analysis (n=624)

Variables	Category	Frequency	Percentage
Mother age (in years)	15-24	88	14.10
	25-34	312	50.00
	35yrs and above	224	35.90
Residence	Rural	450	72.12
	Urban	174	27.88
Religion	Orthodox	575	92.15
	Muslim	49	7.85
Marital status	Married	463	74.2
	Not currently married	161	25.8
Mother	Primary	158	25.32
	Secondary & above	109	17.47

Father	Primary	117	25.27
	Secondary & above	113	24.41
Occupation of Mothers	Farmer	297	47.60
	Housewife	174	27.88
	Government	37	5.93
	Merchant	79	12.66
	Daily laborer	37	5.93
Child age	Above 2 yrs.	128	20.51
	2yrs and under 2yrs	496	79.49
Sex of the child	Male	321	51.44
	Female	303	48.56
	Yes	70	11.22
education about Trachoma	No	554	88.78
	High	243	38.94
illiteracy level	Low	381	61.06

Table 2:-Environmental and other related characteristics of mothers having children aged under nine years in Andabet district, north west Ethiopia,2022 :a multi-level analysis (n=624)

Variables	Category	Frequency	Percent
Source of water	River	457	73.24
	Household tap	167	26.76

Time taken to water Point	<=30 min	282	45.19
	>30 min	342	54.81
Amount of water used per person per day	<=20 liter	128	20.51
	>20 liter	496	79.49
Frequency of getting water	All the time	516	82.69
	Either day or night	17	2.72
	In more than a day	91	14.59
Source of Energy for cooking			
Electricity	Yes	12	1.92
	No	612	98.08
Wood	Yes	615	98.56
	No	9	1.44
Animal dung	Yes	610	97.76
	No	14	2.24
Charcoal	Yes	278	44.62
	No	345	55.38
Type of household latrine used	Covered pit latrine	414	84.49
	Uncovered pit latrine	76	15.51

Cleanness of latrine	Clean	327	67.01
	Not clean	161	32.99
Availability of hand washing material near to latrine	Yes	15	3.07
	No	473	96.93
Cleanness of the home compound	Clean	176	28.21
	Not clean	448	71.79
availability of community latrine	Yes	153	24.52
	No	471	75.48
Basic trachoma knowledge	Good	219	35.1
	Poor	405	64.9
Attitude towards trachoma	Good	354	56.73
	poor	270	
			43.27

Table 3:-Trachoma prevention practice and associated factors among mothers having children aged under nine years in Andabet district, north west Ethiopia ,2022:a multi-level analysis (n=624)

483

Variables	Frequency	Percentage
Mother facial cleanness	427	68.43
Clean		
Not clean	197	31.57
Child facial cleanness		
Clean	167	26.76
Not clean	457	73.24
Using soap for face washing		
Yes	491	78.69
No	133	21.31
Did not share fomites with family		
Yes	460	73.72
No	164	26.28
Separated house for animal dwelling		
Yes	296	47.44
No	328	52.56
Availability of household latrine		

Yes	488	78.21
NO	136	21.79
Infant feces disposal to latrine		
Yes	482	77.24
No	142	22.76
Utilization of latrine		
Yes	483	77.4
No	141	22.6
Availability of waste disposal pit		
Yes	289	46.39
No	334	53.61
Utilization of waste disposal pit		
Yes	293	46.96
No	331	53.04

Table 4:-random effect in trachoma prevention practice among mothers having children aged under nine years in Andabet, North west Ethiopia,2022:a multi-level analysis (n=624)

Parameter	Model 1	Model 2	Model 3	Model 4
MOR	6.51	2.90	3.77	2.67
PCV	Reff.	0.678	0.497	0.706
ICC	0.54	0.27	0.37	0.25
Deviance	583.69	536.13	576.34	531.74

Table 5:-Multilevel logistic regression analysis factors associated with trachoma prevention practice among mothers having children aged under nine years in Andabet district, North west Ethiopia,2022:a multi-level analysis (n=624)

Variables	Model 1	Mode 2 AOR 95%(CI)	Mode 3 AOR 95%(CI)	Mode 4 AOR 95%(CI)
Mother education				
No formal education		2.97(1.42,6.19)		2.95(1.41,6.15)**
Primary		2.36 (1.05, 5.32)		2.33(1.04,5.24)*
Secondary & above		1.00		1.00
Maternal occupation				
Housewife		1.00		1.00
Farmer		3.16(1.82,5.50)		3.02(1.73,5.28) ***
Government employee		1.40(0.45,4.35)		1.39(0.44,4.37)
Merchant				

	2.64(1.21,5.75)	2.63(1.20,5.75) *
Daily laborer		1.61(0.60,4.36)
	1.64(0.61,4.41)	
Frequency of getting water		
All the time	1.00	1.00
Day or night	0.45(0.10,2.32)	0.51(0.10,2.50)
In > a day	0.48(0.2,1.13)	0.50(0.21,1.19)
Time taken to water point		
≤30 minute	1.00	1.00
> 30 minutes	5.85(1.70,20.10)	4.60(1.30,16.26)*
Taking education about trachoma		
No	2.25(1.11,4.57)	2.36(1.16,4.79)*
Yes	1.00	1.00

Source of water		
River	0.45(0.07,3.15)	0.54(0.07,4.26)
Household tap		
	1.00	1.00
Residence		
Rural	3.53(0.53,23.54)	3.16(0.42,23.70)
Urban	1.00	1.00
Community women		
illiteracy	0.09(0.01,0.87)	0.46(0.1,3.55)
Low		
High	1.00	1.00

note: *** =P<0.001, ** P<0.01, and *= P<0.05

Figures

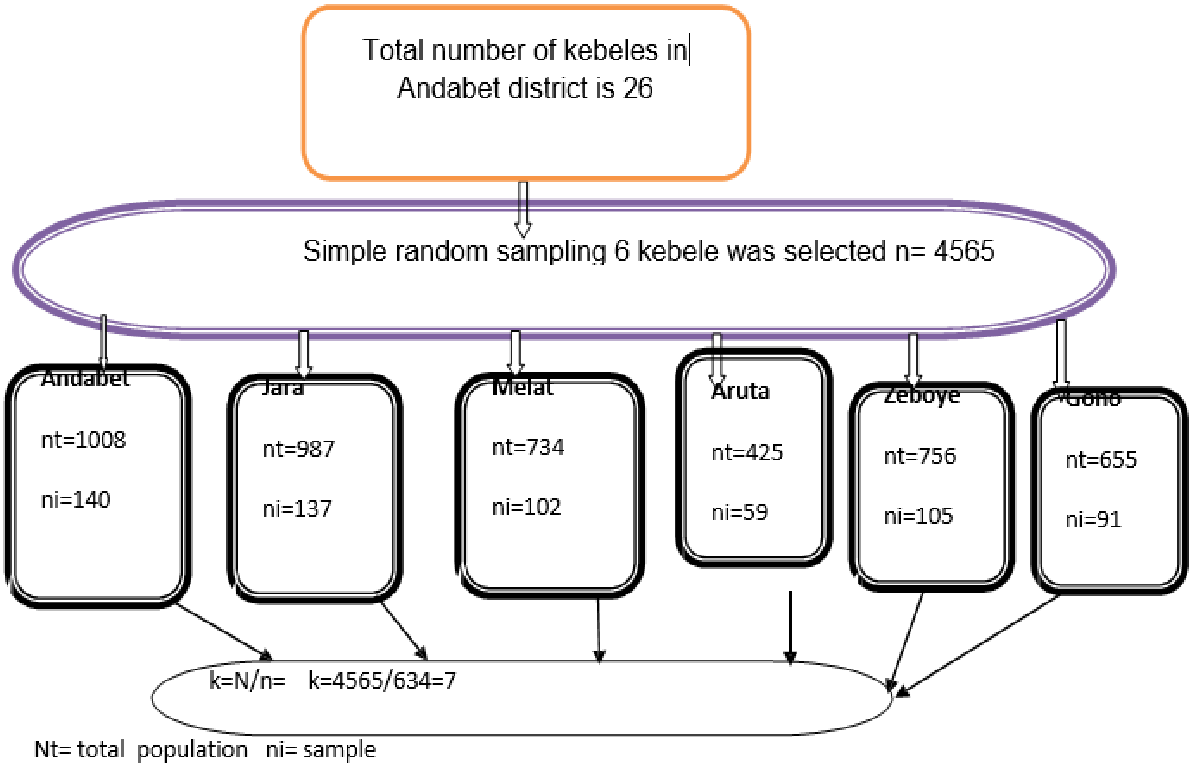

Figure 1:-schematic presentation of sampling technique of trachoma prevention practice among mothers having children aged under nine years in Andabet district, North west Ethiopia,2022:a multi-level analysis

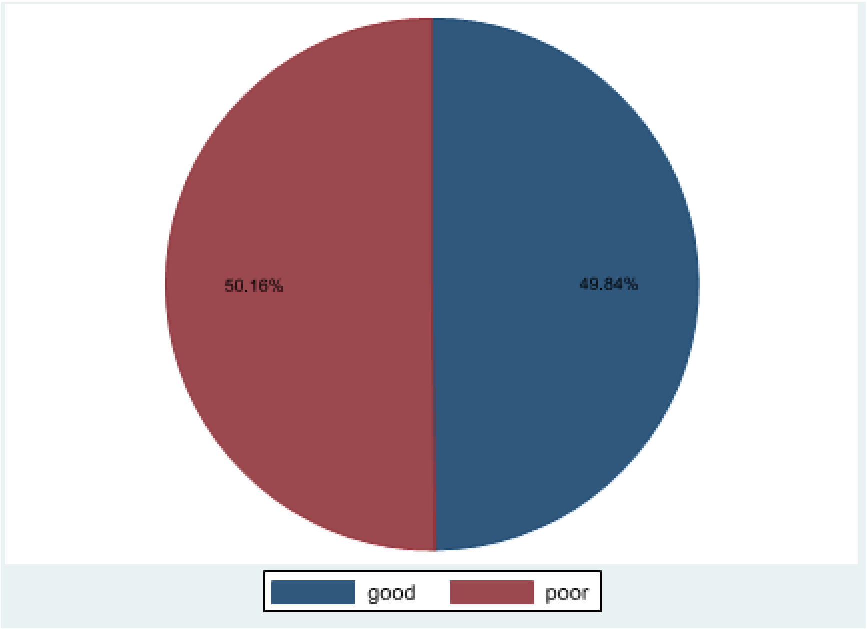

Figure 2: Trachoma prevention practice among mothers having children aged under nine years in Andabet district, North west Ethiopia,2022 :a multi-level analysis (n=624)
