# Supplementary material for: Trachoma prevention practice and associated factors among mothers having children aged under nine years in Andabet district, northwest Ethiopia, 2022: A multi-level analysis
Source: PLoS Negl Trop Dis. 2023 Jun 30;17(6):e0011433. doi: 10.1371/journal.pntd.0011433 (PMC10313038; doi:10.1371/journal.pntd.0011433)
Supplement: Attachment — Submitted filename: PNTD-D-22-01493_R1-1-highlights-comments.pdf [file pntd.0011433.s004.pdf]

PLOS Neglected Tropical Diseases

Trachoma prevention practice and associated factors among mothers having children aged under nine years in Andabet district, northwest Ethiopia, 2022: A multi-level analysis

--Manuscript Draft--

**Manuscript Number:**	PNTD-D-22-01493R1
**Full Title:**	Trachoma prevention practice and associated factors among mothers having children aged under nine years in Andabet district, northwest Ethiopia, 2022: A multi-level analysis
**Short Title:**	Trachoma prevention practice and associated factors among mothers
**Article Type:**	Research Article
**Keywords:**	Key word: Trachoma, Trachoma prevention practice, multi-level analysis, random effect analysis.
**Abstract:**	A b s tract<br>Background<br>The world health organization adopted the Surgery, Antibiotic, facial cleanness, and environmental improvement (SAFE) strategy for the prevention of trachoma, and different prevention strategies have been employed in Andabet district. Trachoma still has a high prevalence despite these efforts. So, it is imperative to assess ground trachoma prevention practices since there are insufficient studies in the study area.<br>Objective<br>To determine the magnitude and factors associated with trachoma prevention practice among mothers having children aged under nine years in Andabet district, Northwest Ethiopia.<br>M e thod<br>A community-based cross-sectional study involving 624 participants was conducted from June 1- 30, 2022. Systematic random sampling was carried out to select study participants. Multi-level binary logistic regression analysis was used to identify factors associated with poor TPP. Descriptive and summary statistics were performed and Variables with p-value < 0.05 in the best-fitted model were declared to be significantly associated with poor TPP.<br>Results<br>In this study, the proportion of poor TPP was found to be 50.16% (95%CI=46.23, 54.08). In the multi-variable multi-level logistic regression; having no formal education (AOR= 2.95; 95%CI: 1.41,6.15) and primary education (AOR= 2.33; 95%CI:1.04, 5.24), being a farmer (AOR=3.02; 95%CI:1.73,5.28), and merchant (AOR=2.63; 95%CI:1.20, 5.75), time taken to water point >30 minutes (AOR=4.60,95CI:1.30,16.26) and didn't take health education about trachoma (AOR=2.36;95CI:1.16,4.79) were significantly associated with poor TPP.<br>Conclusion<br>The proportion of poor trachoma prevention practice was high relative to other studies. Level of education, occupation, time taken to the water source, and health education were significantly associated with poor trachoma prevention practice. Therefore, taking special attention to these high-risk groups could decrease the poor TPP.
**Additional Information:**	
**Question**	**Response**
**Government Employee**<br><br>Are you or any of the contributing authors an employee of the United States government?	No - No authors are employees of the U.S. government.

Powered by Editorial Manager® and ProduXion Manager® from Aries Systems Corporation

Manuscripts authored by one or more US Government employees are not copyrighted, but are licensed under a CC0 Public Domain Dedication, which allows unlimited distribution and reuse of the article for any lawful purpose. This is a legal requirement for US Government employees.<br><br>This will be typeset if the manuscript is accepted for publication.	
**Financial Disclosure**<br><br>Enter a financial disclosure statement that describes the sources of funding for the work included in this submission and the role the funder(s) played. This includes grants and any commercial funding of the work or authors.<br><br>This statement will be typeset if the manuscript is accepted for publication.<br><br>Review the submission guidelines and the instructions link below for detailed requirements and guidance.	The data collection was financially covered by the University of Gondar, Ethiopia. . However,<br>The funder had no role in the study design, data collection, and analysis, decision to publish or the<br>Preparation of the manuscript.
**Competing Interests**<br><br>On behalf of all authors, disclose any competing interests that could be perceived to bias this work.<br><br>This statement will be typeset if the manuscript is accepted for publication.<br><br>Review the instructions link below and PLOS NTDs' competing interests policy to determine what information must be disclosed at submission.	The authors declare that they have no competing interest
**Data Availability**<br><br>Provide a **Data Availability Statement** in the box below. This statement should	The datasets used during the current study are available from the corresponding author upon request.

detail where the data used in this submission can be accessed. This statement will be typeset if the manuscript is accepted for publication.<br><br>Before publication, authors are required to make all data underlying their findings fully available, without restriction. Review our PLOS Data Policy page for detailed information on this policy. Instructions for writing your Data Availability statement can be accessed via the Instructions link below.	

Powered by Editorial Manager® and ProduXion Manager® from Aries Systems Corporation

Manuscript Click here to access/download;Manuscript;Manuscript.docx

Trachoma prevention practice and associated factors among mothers having children aged under nine years in Andabet district, northwest Ethiopia, 2022: A multi-level analysis

Zufan Alamrie Asmare [1*], Natnael Lakachew Assefa [2], Dagmawi Abebe[3], Solomon Gedlu Nigatu [4], Yezinash Addis Alimaw [2]

[1]Department of Ophthalmology, School of Medicine and Health Science, Debre Tabor University, Debre Tabor, Ethiopia

[2]Department of optometry, School of Medicine and Health Science, University of Gondar, Gondar, Ethiopia

[3]Department of Ophthalmology, School of Medicine and Health Science, University of Gondar, Gondar, Ethiopia

[4] Department of Epidemiology & Biostatistics, School of Medicine and Health Science, University of Gondar, Gondar, Ethiopia

***corresponding author**

Zufan Alamrie Asmare (Debre Tabor University School of Medicine and Health Science)

EMAIL: zufanalamrie2@gmail.com

PHONE NO; +251937374268,

P.O BOX- 272

Abstract

Background

The world health organization adopted the Surgery, Antibiotic, facial cleanness, and environmental improvement (SAFE) strategy for the prevention of trachoma, and different prevention strategies have been employed in Andabet district. Trachoma still has a high prevalence despite these efforts. So, it is imperative to assess ground trachoma prevention practices since there are insufficient studies in the study area.

Objective

To determine the magnitude and factors associated with trachoma prevention practice among mothers having children aged under nine years in Andabet district, Northwest Ethiopia.

Method

A community-based cross-sectional study involving 624 participants was conducted from June 1- 30, 2022. Systematic random sampling was carried out to select study participants. Multi-level binary logistic regression analysis was used to identify factors associated with poor TPP. Descriptive and summary statistics were performed and Variables with p-value < 0.05 in the best-fitted model were declared to be significantly associated with poor TPP.

Results

In this study, the proportion of poor TPP was found to be 50.16% (95%CI=46.23, 54.08). In the multi-variable multi-level logistic regression; having no formal education (AOR= 2.95; 95%CI: 1.41,6.15) and primary education (AOR= 2.33; 95%CI:1.04, 5.24), being a farmer (AOR=3.02; 95%CI:1.73,5.28), and merchant (AOR=2.63; 95%CI:1.20, 5.75), time taken to water point >30 minutes (AOR=4.60,95CI:1.30,16.26) and didn't take health education about trachoma (AOR=2.36;95CI:1.16,4.79) were significantly associated with poor TPP.

Conclusion

The proportion of poor trachoma prevention practice was high relative to other studies. Level of education, occupation, time taken to the water source, and health education were significantly associated with poor trachoma prevention practice. Therefore, taking special attention to these high-risk groups could decrease the poor TPP.

Keywords: Trachoma, Trachoma prevention practice, multi-level analysis, random effect analysis.

Author Summary

Trachoma prevention and control strategies have been successful in certain societies, but it has been more difficult in many communities. In Ethiopia, there are 10.2 million cases of trachoma, in which the Amhara region takes the lion's share. Half the global population requiring intervention for trachoma elimination is in the country where some regions/districts have up to 37%TF rate after years of antibiotic treatment. Facial cleanliness & Environmental improvements are critical for sustained progress toward elimination. This study demonstrates the need to consider support for the introduction of those interventions (Fand E) for trachoma elimination in Ethiopia and thus the elimination of an estimated half the global burden. The findings can be used to establish effective public health approaches and implementation of those strategies (F and E) for trachoma prevention and control.

Introduction

Trachoma is caused by *Chlamydia trachomatis*, a neglected tropical disease (1). It has been one of the most debilitating diseases affecting 60 to 90 % of children, especially those under nine years (2, 3). As a devastating disease, Trachoma hinders the performance of children in school and impairs their ability to lead a healthy and productive life (2). Moreover, these ruinous diseases can result in

vision loss, stigma, reduced productivity, and economic loss of US$ 2.9-5.3 billion annually, and half of the global burden of trachoma is shared by Ethiopia solely (4, 5).

Globally, trachoma causes 1.9 million visual impairments and 1.2 million blindness. It is still endemic in 44 countries (6, 7). More than 80% of active trachoma is concentrated in Africa, nearly half is in sub-Saharan Africa (8). In Ethiopia, there are 10.2 million cases of trachoma, in which the Amhara region takes the lion's share (62.6%) (4, 9).

To control the diseases, WHO implemented SAFE (surgery for advanced disease, Antibiotics, facial cleanliness, and Environmental improvement) (10-13). As a result of the implementation, trachoma prevalence has drastically decreased (8, 14). As of 5 October 2022, fifteen countries have been validated as having eliminated the disease as a public health problem (15). Aside from S (surgery) and A (antibiotics), Proper practice of F (facial cleanness) and E (Environmental improvement) is responsible for 58.7% and 37.4% reduction of trachoma prevalence at all ages and in children respectively (16). Therefore, changes in hygiene behavior and improvements in environmental infrastructure are ideal long-term strategies for trachoma control (17).

Despite these, in our study area Andabet, Northwest Ethiopia, After 8 to 11 years of implementation of SAFE, the prevalence of TF (trachomatous follicular) was 37% in 2017. Which is hyperendemic; other than the other 3 similar settings (9, 18).

According to different studies conducted in Ethiopia, the prevalence of poor trachoma prevention practice was high, which ranges between 45.5 and 64.4%(1, 19). shreds of evidence have shown that individual-level factors such as the age of the mother, husband's education, basic knowledge about trachoma, mother's attitude towards trachoma, taking health education about trachoma, time taken to the water point and frequency of getting water and also community-level factors such as residence and types of water source were affected trachoma prevention practice (1, 7, 19-24).

Although numerous studies were done on trachoma prevention practice, most of them did not consider the community-level factors that could affect trachoma prevention practice. Therefore, we aimed to determine the magnitude and associated factors of trachoma prevention practice. Identifying various factors at both individual and community levels can have a key role in implementing policies and programs aimed at minimizing poor trachoma prevention practice.

Methods

Study area and period

This study was conducted in the Andabet district, south Gondar zone, Amhara region, Ethiopia from June 1-30, 2022. The district is located 717 km from Addis Ababa and 150km east of Bahirdar, the capital city of the Amhara region. Its total population is 152,683 and according to 2022 data from the Andabet district administration office; the district has 26 kebeles with 34765 households. It has 1 primary health care center and 2 health posts. Its climate condition is Woinadega

Study design

A community-based cross-sectional design was used with a systematic random sampling method from June 1-30, 2022.

Source and study population

The source and study population of the study were Mothers having children aged under nine years who had been living in the Andabet district, Northwest Ethiopia. Mothers who had at least one child of age less than nine years and those who lived in the district at least for six months were included in the study.

Exclusion criteria

Mothers with mental illness, other serious systemic illnesses, and hearing problems were excluded from the study.

Sample size determination

The sample size was calculated using a single population proportion formula for the proportion of poor trachoma prevention practice. By taking a similar study done in Oromia, Ethiopia with a proportion of (48.5%) (1), 95% confidence level, 5% margin of error, 1.5 design effect, and 10% non-response rate the final sample size for this study was determined to be 634.

Ethical consideration

The study adhered to the tenets of the Declaration of Helsinki and approval was sought and obtained from the Ethical Review Board of the College of Medicine and Health Sciences, University of Gondar. A permission letter was obtained from Andabet district administrative office and written informed consent was obtained from all voluntary participants. The participants were informed of the study would not impose harm on them. There were no personal identifiers and the confidentiality of the study participants was maintained at all stages of data processing. Informed verbal consent was obtained from each respondent and Confidentiality was kept by using codes and avoiding personal identifiers.

Sampling technique and procedure

A multistage sampling technique was used during the sampling process. Six Kebeles out of 26 Kebeles were selected by using a simple random sampling method after a list of kebeles was obtained from the Andabet district administration bureau. The total sample size of the study was allocated proportionally for each Kebele based on the number of mothers having children aged

under nine years that were found in each Kebele. Finally, the households were chosen using a systematic random sample technique.

To carry out systematic random sampling, sampling frames were collected from each kebele. The total estimated number of the study population was 4565. Based on the study population and sample size required from each kebele, we calculated the interval between households and found it to be seven. Then the first household was randomly selected from 1 to 7 serial numbers of sampling and the remaining households were selected at every 7th interval. If mothers in the household did not fulfill the inclusion criteria and/or who did not found with two repeated visits the next household was taken (Figure 1),

Figure 1:-schematic presentation of sampling technique of trachoma prevention practice among mothers having children aged under nine years in Andabet district, Northwest Ethiopia,2022:a multi-level analysis (n=624)

Variables of the study

Dependent variables

The dependent variable was the mother’s trachoma prevention practice.

Independent variables

For this study, the independent variables were classified as individual and community-level variables. The individual level variables were the Age of the mother, Mother's education, Husband's education, Occupation of the mother, Religion, Marital status Number of children under nine years, age of the youngest child, Sex of the youngest child, Basic trachoma Knowledge, Attitude towards

trachoma and Amount of water used per person per day. Community-level variables for this study were Residence, Type of water source, and Community women's illiteracy level.

Operational definition

Trachoma prevention practice assessment: is the assessment of the mother's trachoma prevention practice towards F and E components of SAFE and is classified as good or poor based on the mean of the scores (1).

Basic trachoma Knowledge assessment: is the assessment of the mother's basic knowledge of trachoma and is classified as good or poor based on the mean of the scores (25)

Attitude towards trachoma assessment: this is the assessment of the mother's attitude towards trachoma and is classified as good or poor based on the mean of the scores (25-27).

Facial cleanliness: measured as an absence of ocular discharge, nasal discharge, and fly (s) on the eye during the time of examination. If there was one from the list, considered not clean (28).

Ocular discharge: Any discharge around and/or in the eye at the time of examination (28).

Nasal discharge: any discharge seen in the nose at the time of examination (28).

Fly-eye: at least one fly contact with the eyelid margin during eye observation (29).

Time taken to the water source: collection time does not exceed 30 minutes.

Availability of water: An average person uses about 20 liters of water per day for Domestic and personal hygiene (30).

Utilization of waste disposal pit: Disposal pits that had at least one of the following: discarded unwanted agricultural products, domestic products, or ashes (a burned sign of waste) were considered utilized, otherwise not (31).

Latrine utilization: Latrines that displayed at least two of the following during the observation: footpath to the latrine, fresh excreta inside the latrine, presence of a splash of urine, and the absence of a spider web of the squat were considered utilized, otherwise not (32).

Cleanness of compound: a household (residential) compound free from solid waste, liquid wastes, feces, animal dung, and domestic waste was considered clean (25).

Health education: Those who received education about trachoma and trachoma prevention at least once in the past two years were considered to have taken health education (31).

Cleanness of latrine: if there is at least one of these; human excreta out of the pit, stagnant urine, and unwanted trash on the floor of the latrine, it was not considered clean (33).

Community-women illiteracy: it is the aggregated community-level variable derived from maternal educational level and measured as the proportion of women with no formal education at the kebele/community level. Based on a median value it was then dived into low (mothers from communities with lower illiteracy levels) and high (mothers from communities with higher illiteracy levels) categories (34, 35).

Data collection procedure and quality control

Quantitative data was collected through a face-to-face interview (supported by observation when it is important) by using an interviewer-administered questionnaire Which was adapted from different literature and modified to the context. The questionnaire was first developed in English language and then translated into Amharic (the local language). The questionnaire has six different parts. Part-I: comprising of socio-demographic questions, Part-II: comprises fifteen different knowledge-assessing questions, Part-III: comprises seven different attitude-assessing questions, Part-IV:

comprises ten questions assessing the trachoma preventive practice, Part-V: comprises Environmental related questions and part-VI comprises the observation checklist.

Pretest was done on 5% of the total sample size at zeboye district in the south Gondar zone. After the pretest, necessary modifications and corrections took place to ensure validity. Four data collectors and one supervisor were recruited and trained for 1 day to collect and supervise the data respectively. The reliability of the question for trachoma prevention practice was checked by Cronbach alpha and the scale reliability coefficient was 0.795.

Data processing and Statistical analysis

Data was entered into Epi-Data version 4.6 then data cleaning, coding, and analysis were done using STATA version 16. Descriptive statistics were reported using text, tables, and figures. The proportion of poor trachoma prevention practice with its 95% Confidence interval (CI) was reported. A multilevel logistic regression analysis was used to assess factors associated with trachoma prevention practice to consider the hierarchical nature of the data in which mothers were nested within-cluster and mothers within the same cluster are more likely to share similar characteristics than mothers in another cluster which violates the independent assumptions of the standard logistic regression model such as the independent and equal variance assumptions.

While conducting a multilevel binary logistic regression analysis, we fitted both random effect and fixed effect analyses. The random effect parameter, intraclass correlation coefficient (ICC) quantifies the degree of heterogeneity of trachoma prevention practice between clusters and an ICC of more than 10% indicates that accounting for the cluster-level variability of trachoma preventive practice using multi-level analysis is appropriate. Moreover, proportion change in variance (PCV), and median odds ratio (MOR) were assessed.

In fixed effect analysis, four models were fitted; model 1 (with the outcome variable only), model 2 (incorporating individual-level variables), model 3 (fitted with community-level variables), and

model 4 (incorporating both individual and community-level variables simultaneously). Among the four models fitted, the last model (model 4) was selected as the best-fitted model since it has the lowest deviance and highest PCV. For all models fitted, the adjusted odds ratio (AOR) with its 95% CI was reported. However, the interpretations are based on the final model, the best-fit model.

Both bivariable and multivariable multilevel logistic regression was done and variables with p-value <0.2 in the bivariable analysis were considered multivariable analysis. Finally, variables with p<0.05 in the multivariable multilevel analysis were declared to be significantly associated with trachoma prevention practice.

Result

Socio-demographic characteristics of study participants

A total of 624 study participants were included in the study. With a mean age of 2, the majority (79.49%) of mothers had children aged less than or equal to two years. most, 88.78% of mothers didn't get health education about trachoma while More than half (57.2%) of them did not receive any formal education (Table 1).

Table 1: socio-demographic characteristics of mothers having children aged under nine years in Andabet, northwest Ethiopia, 2022: a multi-level analysis (n=624)

Variables	Category	Frequency	Percentage
Mother's age (in years)	15-24	88	14.10
	25-34	312	50.00
	35yrs and above	224	35.90
Residence	Rural	450	72.12

	Urban	174	27.88
Religion	Orthodox	575	92.15
	Muslim	49	7.85
Marital status	Married	463	74.2
	Not currently married	161	25.8
Educational level Of mother	No formal education	357	57.21
	primary	158	25.32
	Secondary & above	109	17.47
Educational level Of father	No formal education	233	50.32
	primary	117	25.27
	Secondary & above	113	24.41
Occupation of mothers	farmer	297	47.60
	Housewife	174	27.88
	Government employee	37	5.93
	Merchant	79	12.66
	Daily laborer	37	5.93
Child age	Above 2 yrs.	128	20.51
	2yrs and under 2yrs	496	79.49
Sex of the child	male	321	51.44

	female	303	48.56
Taking health Education about trachoma	yes	70	11.22
	No	554	88.78
Community-women Illiteracy level	high	243	38.94
	low	381	61.06

Environmental and related characteristics

Around three-fourths (73.24%) of mothers got water from the river and 342 (54.81%) of them travel more than 30 minutes to get water. Around two third (64.9%) and 354(56.73%) of mothers had poor basic trachoma knowledge and attitude towards trachoma. Regarding cleanness of the compound, 448 (71.79%) of mothers had unclean house compound (Table 2)

Table 2:-Environmental and other related characteristics of mothers having children aged under nine years in Andabet district, northwest Ethiopia,2022:a multi-level analysis (n=624)

Variables	Category	Frequency	Percentage
Source of water	River	457	73.24
	Household tap	167	26.76
Time taken to Water point	<=30 min	282	45.19
	>30 min	342	54.81
Amount of water used per	<=20 liter	128	20.51

Person per day	>20 liter	496	79.49
Frequency of Getting water	All the time	516	82.69
	Either day or night	17	2.72
	In more than a day	91	14.59
Source of energy for cooking			
electricity	Yes	12	1.92
	No	612	98.08
wood	Yes	615	98.56
	No	9	1.44
Animal dung	Yes	610	97.76
	No	14	2.24
Charcoal	Yes	278	44.62
	No	345	55.38
Type of household latrine used	Covered pit latrine	414	84.49
	Uncovered latrine	76	15.51
Cleanness of latrine	Clean	327	67.01
	Not clean	161	32.99
Availability of hand washing	Yes	15	3.07

Material near to latrine	No	473	96.93
Cleanness of the home compound	Clean	176	28.21
	Not clean	448	71.79
Availability of community latrine	Yes	153	24.52
	No	471	75.48
Basic trachoma Knowledge	Good	219	35.1
	Poor	405	64.9
Attitude towards Trachoma	Good	354	56.73
	Poor	270	43.27

The magnitude of Trachoma prevention practice

In this study 49.84 %(95%CI: 45.91%, 53.76%) of trachoma prevention practice was good and 50.16 % (95%CI: 46.23%, 54.08%) trachoma prevention practice was poor (Figure 2). More than two third of mothers had a clean face, 427 (68.43%), and three fourth of children in the study had an unclean face, 457 (73.24%). More than three fourth (77.40%) of the mothers use latrines (Table 3).

Table 3:-Trachoma prevention practice and associated factors among mothers having children aged under nine years in Andabet district, northwest Ethiopia,2022:a multi-level analysis (n=624)

Variables	Category	Frequency	Percentage

Mother facial cleanness	Clean	427	68.43
	Not clean	197	31.57
Child facial cleanness	Clean	167	26.76
	Not clean	457	73.24
Using soap for face washing	Yes	491	78.69
	No	133	21.31
Did not share fomites With family	Yes	460	73.72
	No	164	26.28
Separated house for Animal dwelling	Yes	296	47.44
	No	328	52.56
Availability of household Latrine	Yes	488	78.21
	No	136	21.79
Infant feces disposal To latrine	Yes	482	77.24
	No	142	22.76
Utilization of latrine	Yes	483	77.40
	No	141	22.60
Availability of waste Disposal pit	Yes	289	46.39
	No	334	53.61

Utilization of waste Disposal pit	Yes	293	46.96
	No	331	53.04

Figure 2: Trachoma prevention practice among mothers having children aged under nine years in Andabet district, Northwest Ethiopia,2022:a multi-level analysis (n=624)

Random effect and model comparison

In the random effect analysis, in the null model, about 54% of the total variation in trachoma prevention practice occurred at the cluster level and is attributable to community-level factors. In addition, the null model also had the highest MOR value (6.51) indicating when randomly selecting a mother from one kebele with a higher risk of poor trachoma prevention practice and the other kebele at lower risk, mothers at the cluster (kebele) with a higher risk of poor trachoma prevention practice had 6.51 times higher odds of having a poor trachoma prevention practice as compared with their counterparts. Furthermore, the highest PCV (70.4%) in the final model (model 4) showed 70.4% of the variation in trachoma prevention practice across communities was explained by both individual and community-level factors. The model fitness was checked by using deviance and the model with the lowest deviance (model4) was the best-fitted model (Table 4).

Table 4:-random effect analysis in trachoma prevention practice among mothers having children aged under nine years in Andabet, Northwest Ethiopia,2022 (n=624)

Parameter	Model 1	Model 2	Model 3	Model 4
MOR	6.51	2.90	3.77	2.67
PCV	Reff.	0.678	0.497	0.706
ICC	0.54	0.27	0.37	0.25
Deviance	583.69	536.13	576.34	531.74

Factors associated with trachoma prevention practice

In the multivariable multi-level logistic regression, maternal education level, maternal occupation, time taken to the water point, and health education about trachoma were significantly associated with poor trachoma prevention practice.

Mothers with no formal education had 2.95(AOR=2.95; 95%CI:1.41,6.15) times higher odds of poor trachoma prevention practice as compared to those mothers with secondary education or above. Mothers with primary education had 2.33 (AOR= 2.33; 1.04,5.24) times higher odds of poor trachoma prevention practice as compared to those mothers with secondary education or above. on maternal occupation, Mothers who were farmers had 3.02(AOR=3.02; 95%CI:1.73, 5.28) times higher odds of poor trachoma prevention practice as compared to those mothers who were housewives. Mothers who were merchants had 2.63 (AOR=2.63; 95%CI:1.20, 5.75) times higher odds of poor trachoma prevention practice as compared to those mothers who were housewives. Regarding time taken to the water point, mothers who traveled >30 minutes to the water point had 4.60 (AOR= 4.60, 95CI:1.30, 16.26) times higher odds of poor trachoma prevention practice as compared to those mothers who traveled ≤30 minutes. Mothers who didn't take health education about trachoma had 2.36 (AOR= 2.36; 95%CI:1.16, 4.79) times higher odds of poor trachoma prevention practice compared to their counterparts (Table 5).

Table 5:-Multilevel logistic regression analysis factors associated with trachoma prevention practice among mothers having children aged under nine years in Andabet district, Northwest Ethiopia,2022:a multi-level analysis (n=624)

variables	Model 1	Mode 2 AOR 95%(CI)	Mode 3 AOR 95%(CI	Mode 4 AOR 95%(CI)

Maternal education				
No formal education		2.97(1.42,6.19)		2.95(1.41,6.15)**
Primary		2.36 (1.05, 5.32)		2.33(1.04,5.24)*
Secondary & above		1.00		1.00
Maternal occupation				
housewife		1.00		1.00
farmer		3.16(1.82,5.50)		3.02(1.73,5.28) ***
Government employee		1.40(0.45,4.35)		1.39(0.44,4.37)
Merchant		2.64(1.21,5.75)		2.63(1.20,5.75) *
Daily laborer		1.64(0.61,4.41)		1.61(0.60,4.36)
Frequency of getting water				
All the time		1.00		1.00
Day or night		0.45(0.10,2.32)		0.51(0.10,2.50)
In > a day		0.48(0.2,1.13)		0.50(0.21,1.19)
Time taken to water source				

≤30 minute		1.00		1.00
> 30 minutes		5.85(1.70,20.10)		4.60(1.30,16.26)*
Taking education about trachoma				
No		2.25(1.11,4.57)		2.36(1.16,4.79)*
Yes		1.00		1.00
Source of water				
River			0.45(0.07,3.15)	0.54(0.07,4.26)
Household tap			1.00	1.00
Residence				
Rural			3.53(0.53,23.54)	3.16(0.42,23.70)
Urban			1.00	1.00
Community-women illiteracy				
Low			0.09(0.01,0.87)	0.46(0.1,3.55)
High			1.00	1.00

Note: *** =P<0.001, ** P<0.01, and *= P<0.05

Discussion

The study aimed to assess the magnitude and associated factors of trachoma prevention practice in the andabet district, Northwest Ethiopia. According to the finding of this study, the magnitude of poor trachoma prevention practice was 50.16%. This finding is in line with a study conducted in Oromia Ethiopia (1). However, this magnitude of poor trachoma prevention practice was found lower compared to a study conducted in Tigray (25) and higher than a study conducted in north Vietnam and the Lemo district of southern Ethiopia (36, 37). The discrepancy might be due to the difference in the study population in which most of the indicated studies (except the study in Oromia, Ethiopia, 2021) children under nine years were their study subjects. Besides most of the above studies were based on small sample size. The other possible explanation is due to the study period and different in availability and accessibility of maternal health services and facilities. Moreover, the discrepancy of this finding with that of the findings of studies conducted out of Ethiopia might be due to socio demographic and cultural differences.

The study at hand found that Mothers with no formal education and mothers with primary education are more likely to have poor prevention practice as compared with those mothers with secondary or above. This is supported by a study done in Vietnam (38). which similarly showed that those with no formal education are more likely to have poor prevention practice. This might be due to the levels and ways of understanding regarding the mechanism of transmission, prevention measures, and negative effects of the diseases being different among mothers with different levels of education. That is educated mothers would likely appreciate the problems related to poor prevention practice more than those with no formal education (36, 39).

In this study, health education is another important variable significantly associated with trachoma prevention practice. That is mothers who didn't take health education about trachoma were more likely to have poor trachoma prevention practice as compared to their counterparts. This finding is supported by a study conducted in the lemo district (37). Such a correlation could be because mothers who have not attended health education programs lack the skills needed to prevent

trachoma. Hence, they are more likely to have cultural misconceptions about how to use water for environmental sanitation and personal hygiene (40, 41).

Moreover in this study mothers who were farmers and merchants were more likely to have poor trachoma prevention practice as compared to those mothers who were housewives. It might be because based on our study 88% of farmers and 93.6% of merchants did not attend health education programs on trachoma.in addition, most (81.5%) of them have to travel more than 30 minutes to get water making it more difficult for them to clean their face and improve their environment than those who are housewives.

Consistent with other studies conducted in Kenya and Oromia (1, 42), in this study, time taken to the water point is significantly associated with trachoma prevention practice; those mothers who traveled more than 30 minutes to the water point had higher odds of poor trachoma prevention practice as compared to their counterparts. This might be due to access to and adequacy of water differs between mothers who travel over 30 minutes and those who travel less than 30 minutes Mothers who have insufficient water may not be able to use it for facial and environmental cleanliness. Furthermore, based on our study, the majority (81.5%) of mothers traveling over 30 minutes for water get it from unclean streams. while most (74%) of those mothers traveling less than 30 minutes get it from relatively clean personal and public pipes (42).

Strengths and limitations of the study

There were strengths and shortcomings in this study. To begin with the strength, this study explored neglected tropical diseases that became hyper-endemic in our study area after the implementation of SAFE for about 8 to 11 years, So the result of the study would be important for employing combined efforts to identified modifiable risk factors and will have significant policy implications in providing support to the affected community besides, the study uses multi-level modeling taking into account the clustering effect to draw valid inference and conclusion.

Moreover, to ensure representativeness, the study uses an adequate sample size. However, this study had limitations as it's a cross-sectional study, It may not show a true temporal relationship between the outcome and the independent variables. Besides, Due to the lack of sign language-trained data collectors and the inability to obtain psychiatric therapists as data collectors, it was not possible to include those mothers with mental illness and hearing problems, although they were quite insignificant. Moreover, the study mainly relies on the mother's self-report, so there may be a chance of recall bias. Furthermore, Social desirability bias might be introduced while assessing sensitive variables

Conclusion

In this study, the magnitude of poor trachoma prevention practice was high relative to other studies. Those mothers with no formal education, with primary education, those who take more time to water point (>30 minutes), those who didn't take health education about trachoma, and those who were farmers and merchants were at higher odds of poor trachoma prevention practice. Therefore, special attention could be taken to these high-risk groups. by doing so this devastating health problem could be decreased.

Recommendations

To governmental and non-governmental organizations: focus on facial cleanness (F) and Environmental improvement (E) components of SAFE.

To Amhara region trachoma control program: planning health education program and enhancing water supply are recommended to improve trachoma prevention practice overall.

To the health office in Andabet district: special attention should be given to those mothers with no formal education and primary education and who are away from a water source. Besides, health education programs about trachoma should be strengthened.

To the global trachoma community: The low coverage in “Trachoma Prevention Practice” will affect WHO launched global alliance for the illumination of blinding Trachoma. Therefore, it is recommended to give special attention to those communities regarding the Facial cleanliness (F) and Environmental Improvement (E) components of the SAFE strategy.

Abbreviations

AOR: adjusted odds ratio; CI: confidence interval; SAFE: Surgery, antibiotics, facial cleanliness, and environmental improvement; TF: trachomatous follicular; ICC: Intra-class Correlation Coefficient; MOR: Median Odds Ratio; PCV: Proportionate Change in Variance; PI: Principal Investigator; TPP: Trachoma prevention practice; WHO: World Health Organization

Supporting information

The datasets used during the current study are available from the corresponding author upon request.

Acknowledgments

We are grateful to thank the Department of Optometry, study participants, and data collectors.

Funding

The data collection was financially covered by the University of Gondar, Ethiopia. . However, The funder had no role in the study design, data collection, and analysis, decision to publish or the Preparation of the manuscript.

References

1. Abera T, Tilahun W, Waqjira I. Trachoma prevention practice among mothers with child age of under-9 years and factors associated in rural district of Oromia Region, Ethiopia:

Community based cross-sectional study. World Journal of Advanced Research and Reviews. 2021;10(1):245-57.
2. Frick KD, Hanson CL, Jacobson GA. Global burden of trachoma and economics of the disease. The American journal of tropical medicine and hygiene. 2003;69(5_suppl_1):1-10.
3. ETAT. Trachoma - a women and children's health issue. 2020.
4. Stewart AE, Zerihun M, Gessese D, Melak B, Sata E, Nute AW, et al. Progress to eliminate trachoma as a public health problem in Amhara National Regional State, Ethiopia: results of 152 population-based surveys. The American journal of tropical medicine and hygiene. 2019;101(6):1286.
5. Bamani S, King JD, Dembele M, Coulibaly F, Sankara D, Kamissoko Y, et al. Where do we go from here? Prevalence of trachoma three years after stopping mass distribution of antibiotics in the regions of Kayes and Koulikoro, Mali. PLoS neglected tropical diseases. 2010;4(7):e734.
6. Greenland K, White S, Sommers K, Biran A, Burton MJ, Sarah V, et al. Selecting behaviour change priorities for trachoma 'F'and 'E'interventions: A formative research study in Oromia, Ethiopia. 2019;13(10):e0007784.
7. Golovaty I, Jones L, Gelaye B, Tilahun M, Belete H, Kumie A, et al. Access to water source, latrine facilities and other risk factors of active trachoma in Ankober, Ethiopia. 2009;4(8):e6702.
8. Stewart AE, Zerihun M, Gessese D, Melak B, Sata E, Nute AW, et al. Progress to eliminate trachoma as a public health problem in Amhara National Regional State, Ethiopia: results of 152 population-based surveys. 2019;101(6):1286.
9. Nash SD, Astale T, Nute AW, Bethea D, Chernet A, Sata E, et al. Population-based prevalence of Chlamydia trachomatis infection and antibodies in four districts with varying levels of trachoma endemicity in Amhara, Ethiopia. The American journal of tropical medicine and hygiene. 2021;104(1):207.
10. Njomo DW, Karimurio J, Odhiambo GO, Mukuria M, Wanyama EB, Rono HK, et al. Knowledge, practices and perceptions of trachoma and its control among communities of Narok County, Kenya. Tropical diseases, travel medicine and vaccines. 2016;2(1):1-10.
11. Aragie S, Wittberg DM, Tadesse W, Dagnew A, Hailu D, Chernet A, et al. Water, sanitation, and hygiene for control of trachoma in Ethiopia (WUHA): a two-arm, parallel-group, cluster-randomised trial. The Lancet Global Health. 2022;10(1):e87-e95.
12. Organization WH. Water sanitation & hygiene for accelerating and sustaining progress on neglected tropical diseases: a global strategy 2015-2020. World Health Organization, 2015.
13. Chikwanda M, Munyinda N, Mwale C, Mbanefo P, Banda TC, Mubita P. An association between water, sanitation, and hygiene (WASH) and prevalence of trachoma in Monze district of Southern Province, Zambia. Journal of Water, Sanitation and Hygiene for Development. 2021;11(3):453-60.
14. Njomo DW, Karimurio J, Odhiambo GO, Mukuria M, Wanyama EB, Rono HK, et al. Knowledge, practices and perceptions of trachoma and its control among communities of Narok County, Kenya. 2016;2(1):1-10.
15. Renneker KK, Abdala M, Addy J, Al-Khatib T, Amer K, Badiane MD, et al. Global progress toward the elimination of active trachoma: an analysis of 38 countries. The Lancet Global Health. 2022;10(4):e491-e500.
16. Khandekar R, Thanah TTK, Thi PD. Impact of face washing and environmental improvement on reduction of active trachoma in Vietnam—a public health intervention study. Ophthalmic Epidemiology. 2006;13(1):43-52.
17. Ejere HO, Alhassan MB, Rabiu M. Face washing promotion for preventing active trachoma. Cochrane Database of Systematic Reviews. 2015(2).
18. Lavett DK, Lansingh VC, Carter MJ, Eckert KA, Silva JC. Will the SAFE strategy be sufficient to eliminate trachoma by 2020? Puzzlements and possible solutions. The Scientific World Journal. 2013;2013.

19. Gebretnsae H, Mamo N, Teklemariam T, Fenta K, Gebrehiwet T, Berhe A, et al. Knowledge, attitudes, and practices about trachoma in rural communities of Tigray Region, Northern Ethiopia: implications for prevention and control. 2020;2020.
20. Asfaw M, Zolfo M, Negussu N, Tadesse F, Tadele T, Sisay A, et al. Towards the trachoma elimination target in the Southern region of Ethiopia: How well is the SAFE strategy being implemented? The Journal of Infection in Developing Countries. 2020;14(06.1):3S-9S.
21. Aiemjoy K, Stoller NE, Gebresillasie S, Shiferaw A, Tadesse Z, Sewnet T, et al. 'If an eye is washed properly, it means it would see clearly': a mixed methods study of face washing knowledge, attitudes, and behaviors in rural Ethiopia. 2016;10(10):e0005099.
22. Lavett DK, Lansingh VC, Carter MJ, Eckert KA, Silva JCJTSWJ. Will the SAFE strategy be sufficient to eliminate trachoma by 2020? Puzzlements and possible solutions. 2013;2013.
23. West SK, Nanji AA, Mkocha H, Munoz B, Gaydos C, Quinn TCJPntd. Evidence for contamination with C. trachomatis in the household environment of children with active Trachoma: A cross-sectional study in Kongwa, Tanzania. 2019;13(12):e0007834.
24. MuluneshG M, Mohammed S,Minichil B. trahoma prevention practice and associated factors in rural lemo district. 2021.
25. Gebretnsae H, Mamo N, Teklemariam T, Fenta K, Gebrehiwet T, Berhe A, et al. Knowledge, attitudes, and practices about trachoma in rural communities of Tigray Region, Northern Ethiopia: implications for prevention and control. Journal of environmental and public health. 2020;2020.
26. Hassali MA, Shafie AA, Saleem F, Farooqui M, Aljadhey H. A cross sectional assessment of knowledge, attitude and practice towards Hepatitis B among healthy population of Quetta, Pakistan. BMC public health. 2012;12(1):1-8.
27. AlRasheed MM, Alsugair AM, Almarzouqi HF, Alonazi GK, Aleanizy FS, Alqahtani FY, et al. Assessment of knowledge, attitude, and practice of security and safety workers toward the COVID-19 pandemic: a cross-sectional Study. Frontiers in public health. 2021:254.
28. Stocks ME, Ogden S, Haddad D, Addiss DG, McGuire C, Freeman MC. Effect of water, sanitation, and hygiene on the prevention of trachoma: a systematic review and meta-analysis. PLoS Med. 2014;11(2):e1001605.
29. Golovaty I, Jones L, Gelaye B, Tilahun M, Belete H, Kumie A, et al. Access to water source, latrine facilities and other risk factors of active trachoma in Ankober, Ethiopia. PLoS One. 2009;4(8):e6702.
30. Tadesse B, Worku A, Kumie A, Yimer SA. Effect of water, sanitation and hygiene interventions on active trachoma in North and South Wollo zones of Amhara Region, Ethiopia: A Quasi-experimental study. PLoS neglected tropical diseases. 2017;11(11):e0006080.
31. MuluneshG M MS, Minichil B. Trahoma prevention practice and associated factors in rural lemo district. 2021.
32. D A. Latrine Utilization and Associated Factors Among Rural Households in Dega Damot Woreda, West Gojjam Zone, Amhara Region, Ethiopia 2021.
33. T. MK. National guideline for water,sanitation and hygine for tanzania school. july,2016.
34. Teshale AB, Tesema GA, Tessema ZT. Spatial variations and associated factors of knowledge of ORS packet or pre-packaged liquids for the management of diarrhea among women of reproductive age in Ethiopia: A spatial and multilevel analysis. PloS one. 2021;16(3):e0247772.
35. Shah AR, Subramanyam MA. Community-level women's education and undernutrition among Indian adolescents: A multilevel analysis of a national survey. PloS one. 2021;16(5):e0251427.
36. Khandekar R, Bullard M, Thanh TTK, Binh TQ. Knowledge and practice related to trachoma among children in Vietnam: A cross-sectional study. Journal of health communication. 2004;9(1):77-83.
37. MuluneshG M MS, Minichil B. Trahoma prevention practice and associated factors in rural lemo district. 2021.

38. Khandekar R, Bullard M, Thanh TTK, Binh TQJJohc. Knowledge and practice related to trachoma among children in Vietnam: A cross-sectional study. 2004;9(1):77-83.

39. david.K. Water Supply and Sanitation in Vietnam. 2 JMP-UNICEF special tabulation per wealth quintile, using MICS 2013.

40. Kuper H, Solomon AW, Buchan J, Zondervan M, Foster A, Mabey D. A critical review of the SAFE strategy for the prevention of blinding trachoma. The Lancet Infectious diseases. 2003;3(6):372-81.

41. Asaolu SO, Ofoezie IE. The role of health education and sanitation in the control of helminth infections. Acta tropica. 2003;86(2-3):283-94.

42. PN. M. . Knowledge, Practices and Perceptionss of Trachoma and its' Influence (Association With) on Health Seeking Behaviour of Patients in Kajiado Central Sub County, Kajiado County, Kenya: COHES, JKUAT; . 2017.

Figure
Click here to access/download;Figure;Figure1.tif

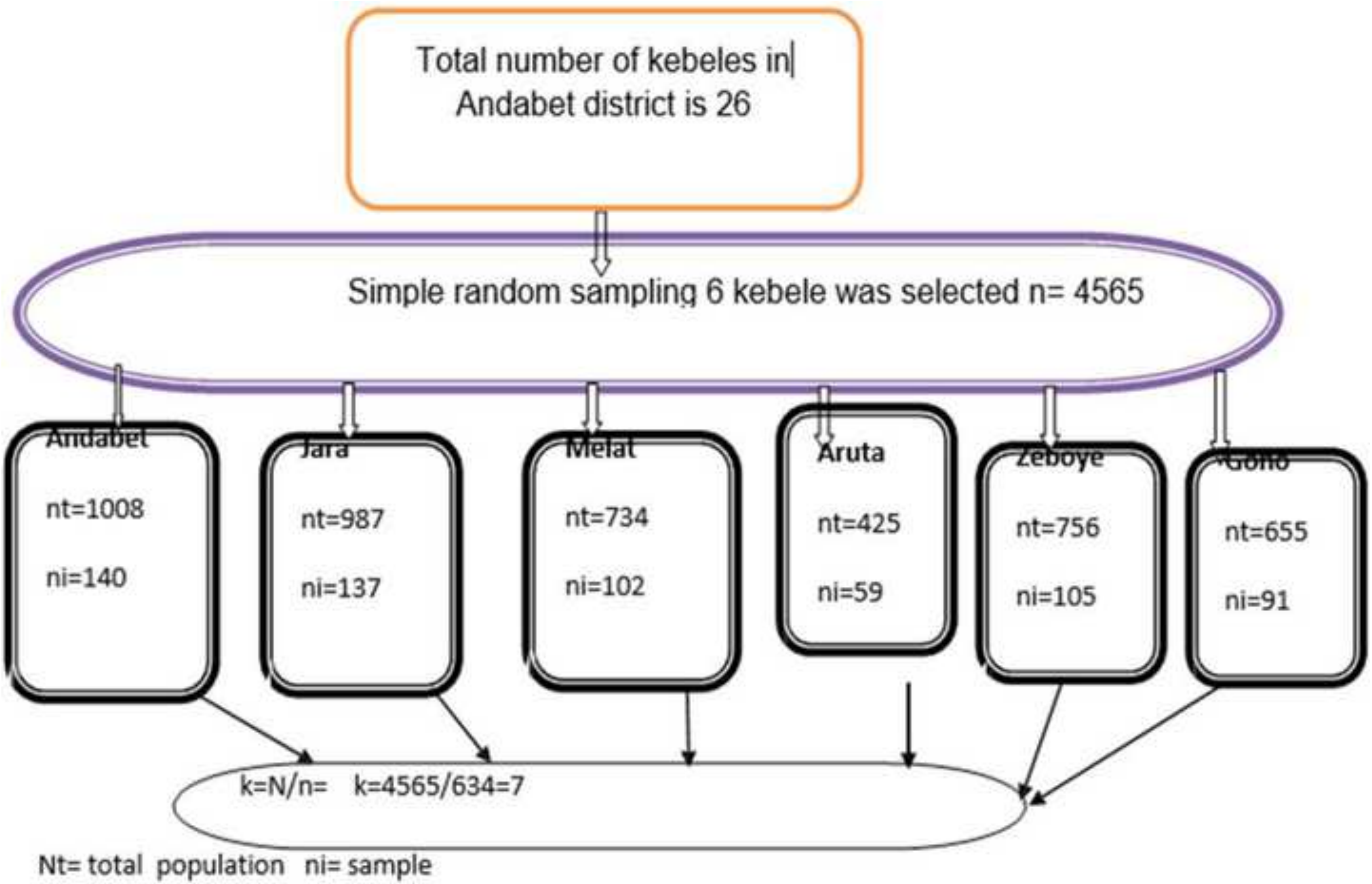

Figure

Click here to access/download;Figure;Figure 2.tif

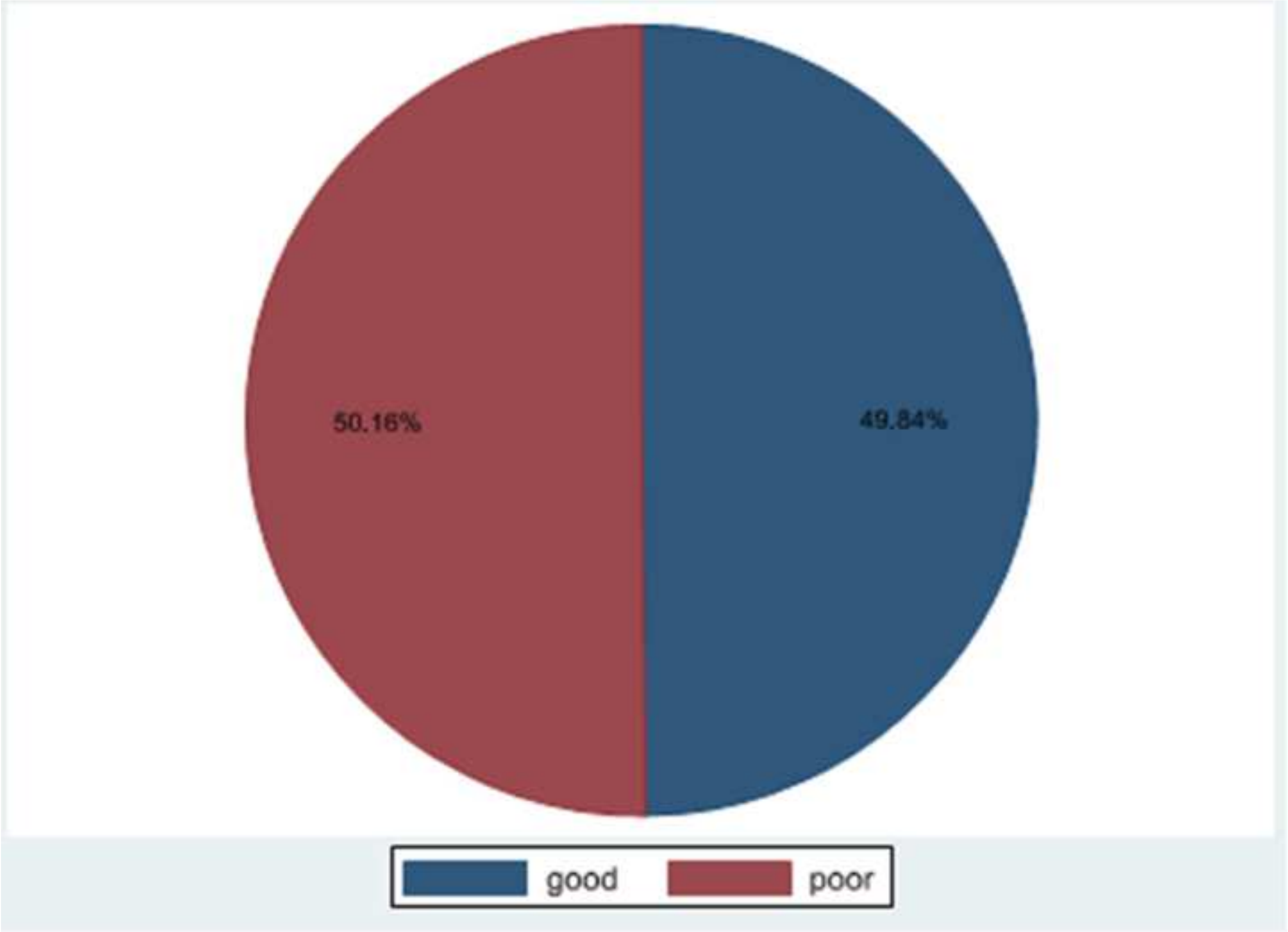

Authors' response to Editor's and Reviewer's comments

The title "Trachoma prevention practice and Associated Factors among mothers having children aged under nine Years in Andabet District, northwest Ethiopia, 2022:a multilevel analysis"

Dear Editor, we have revised our manuscript based on the reviewer's suggestions and comments. Here is a response to the reviewer's and your comments and suggestions

Author's response to the editor's suggestions/comments

When you are ready to resubmit, please upload the following:

[1] A letter containing a detailed list of your responses to the review comments and a description of the changes you have made in the manuscript. Please note while forming your response, if your article is accepted, you may have the opportunity to make the peer review history publicly available. The record will include editor decision letters (with reviews) and your responses to reviewer comments. If eligible, we will contact you to opt-in or out.

Author Response: thank you, dear editor, we have prepared both the letter containing a detailed list of our responses to the review comments and a description of the changes we have made in the manuscript.

[2] Two versions of the revised manuscript: one with either highlights or tracked changes denoting where the text has been changed; the other a clean version (uploaded as the manuscript file).

Author Response: we have prepared the two versions of the manuscript one with tracked change and the other a clean version

Figure Files:

While revising your submission, please upload your figure files to the Preflight Analysis and Conversion Engine (PACE) digital diagnostic tool, https://pacev2.apexcovantage.com. PACE helps ensure that figures meet PLOS requirements. To use PACE, you must first register as a user. Then, login and navigate to the UPLOAD tab, where you will find detailed instructions on how to use the tool. If you encounter any issues or have any questions when using PACE, please email us at figures@plos.org.

Author Response: thank you, dear editor, we have uploaded the figure with PACE

Data Requirements:

Please note that, as a condition of publication, PLOS' data policy requires that you make available all data used to draw the conclusions outlined in your manuscript. Data must be deposited in an appropriate repository, included within the body of the manuscript, or uploaded as supporting information. This includes all numerical values that were used to generate graphs, histograms etc.. For an example see here: http://www.plosbiology.org/article/info%3Adoi%2F10.1371%2Fjournal.pbio.1001908#s5.

Author Response: thank you**,** dear editor the datasets used during the current study are available from the corresponding author upon request.

Response to the reviewer's comment

Dear reviewer, we are pleased and appreciate your vigilant and thorough reading of this manuscript and we would like to say thank you for your thoughtful and constructive comments and

suggestions. Based on your comments and suggestions, we had carefully corrected the manuscript one by one. And the whole document had revised.

Reviewer #1: The study is very important for the global trachoma community for decision-making at global/international, national, regional, district, and community levels by all stakeholders involved as Ethiopia currently has an estimated 50% of the global population requiring interventions for trachoma elimination. Addressing the trachoma problem in Ethiopia is potentially wiping out half the global trachoma problem. There is enough information that shows that the study is important and worthy of publication. However, major revision is needed. The overall presentation is poor. Grammar is poor. I suggest the authors find someone who can proofread the many grammatic mistakes.

Author Response: thank you very much, dear reviewer, a critical revision has been made to the presentation of the revised manuscript, please see the revised version

1. Objectives should be revised. See my comments in the attached file. They are not clearly articulated.

Author Response: thank you, dear reviewer, we have notably improved the objective part of the manuscript .please see the revised version

I believe that the study design is appropriate to address the stated objectives.

The population is clearly described and appropriate for the hypothesis being tested.

The sample size looks sufficient to ensure adequate power to address the hypothesis being tested.

I believe that correct statistical analysis was used to support conclusions although I may not be strong enough on statistical methods.

2. Selection of participants is generally ethical and meets regulatory requirements. However, some women were excluded with weak justification for the exclusion. Better explanation needed in the limitation section.

Author Response: thank you, dear reviewer, we have included the explanation in the limitation part of the revised manuscript. , Due to the lack of sign language-trained data collectors and the inability to obtain psychiatric therapists as data collectors, it was not possible to include those mothers with mental illness, hearing problems and a mother having a severe systemic illness prevents her from answering our question but their number was quite insignificant. It was only 4 mothers excluded from the study based on our exclusion criteria.

3. Reviewer #1: The study is a replication of similar studies conducted in other parts of the country and in other countries. The analysis matches the analysis plan. Results are completely presented. The authors need to improve the presentation in general

Author Response: thank you, dear reviewer, although there are similar studies conducted, this study explored neglected tropical diseases that became hyper-endemic in our study area after the implementation of SAFE for about 8 to 11 years. Other than the three similar settings, in which SAFE was equally implemented, only our study area remained hyper-endemic having a prevalence of 37 %, it remained a mystery. so we were very interested to study the prevention practice of the community and thus the elimination of an estimated half the global burden moreover, this study uses multi-level modeling taking into account the clustering effect to draw valid inferences and conclusions. Hence this study would be important for employing combined efforts to identified modifiable risk factors and will have significant policy implications in providing support to the affected community.

Dear reviewer, we have improved the presentation critically. please see the revised version

4. Reviewer #1: Conclusions are supported by the data presented but presentation is generally poor.

The limitations of analysis are described but more work needed.

The authors tried to discuss how these data can be helpful to advance our understanding of the topic under study, but more work is needed to improve the presentation of the study.

Authors have poorly addressed the public health relevance of the study. I have made some suggestions in the attached file.

Author Response: thank you, dear reviewer, we have critically improved the conclusion, and limitation and addressed the public health relevance of the study in the strength part of the study in the revised version.

5. Reviewer #1: The study is a replication of studies conducted in other countries and in other parts of Ethiopia to assess community practices that influence trachoma prevention and elimination. Half the global population requiring intervention for trachoma elimination is in Ethiopia where some regions/districts have up to 37% TF rate (hyperendemicity) after years of A treatment. This study demonstrates the need to consider support for the introduction of other interventions (F and E) for trachoma elimination in Ethiopia and thus elimination of an estimated half the global burden. I believe that the essential information needed (objectives, methodology, result) is available in this draft of the manuscript. However, overall presentation is poor with too many grammatic errors. Authors should take time to improve the presentation including finding someone who can proofread and edit the manuscript for them. I have made some suggestions in the attached file.

Author Response: thanks a lot dear reviewer, we have critically improved the newly revised manuscript as a whole one by one please look the revised manuscript.

Click here to access/download
Revised Article with Changes Highlighted
revised manuscript with track change.docx
