# Supplementary material for: Trachoma prevention practice and associated factors among mothers having children aged under nine years in Andabet district, northwest Ethiopia, 2022: A multi-level analysis
Source: PLoS Negl Trop Dis. 2023 Jun 30;17(6):e0011433. doi: 10.1371/journal.pntd.0011433 (PMC10313038; doi:10.1371/journal.pntd.0011433)
Supplement: Attachment — Submitted filename: response for reviewer comment.docx [file pntd.0011433.s003.docx]

**Authors’ response to Editor’s and Reviewer’s comments**

**The title “Trachoma prevention practice and Associated Factors among mothers having children aged under nine Years in Andabet District, northwest Ethiopia, 2022:a multilevel analysis”**

Dear Editor, we have revised our manuscript based on the reviewer's suggestions and comments.  Here is a response to the reviewer's and your comments and suggestions

**Author's response to the editor’s suggestions/comments**

When you are ready to resubmit, please upload the following:

[1] A letter containing a detailed list of your responses to the review comments and a description of the changes you have made in the manuscript. Please note while forming your response, if your article is accepted, you may have the opportunity to make the peer review history publicly available. The record will include editor decision letters (with reviews) and your responses to reviewer comments. If eligible, we will contact you to opt-in or out.

**Author Response:** thank you, dear editor, we have prepared both the letter containing a detailed list of our responses to the review comments and a description of the changes we have made in the manuscript.

[2] Two versions of the revised manuscript: one with either highlights or tracked changes denoting where the text has been changed; the other a clean version (uploaded as the manuscript file).

**Author Response:** we have prepared the two versions of the manuscript one with tracked change and the other a clean version

Figure Files:

While revising your submission, please upload your figure files to the Preflight Analysis and Conversion Engine (PACE) digital diagnostic tool, https://pacev2.apexcovantage.com. PACE helps ensure that figures meet PLOS requirements. To use PACE, you must first register as a user. Then, login and navigate to the UPLOAD tab, where you will find detailed instructions on how to use the tool. If you encounter any issues or have any questions when using PACE, please email us at [figures@plos.org](mailto:figures@plos.org).

**Author Response:** thank you, dear editor, we have uploaded the figure with PACE

Data Requirements:

Please note that, as a condition of publication, PLOS' data policy requires that you make available all data used to draw the conclusions outlined in your manuscript. Data must be deposited in an appropriate repository, included within the body of the manuscript, or uploaded as supporting information. This includes all numerical values that were used to generate graphs, histograms etc.. For an example see here: <http://www.plosbiology.org/article/info%3Adoi%2F10.1371%2Fjournal.pbio.1001908#s5>.

**Author Response: th**ank you**,** dear editor the datasets used during the current study are available from the corresponding author upon request.

**Response to the reviewer's comment**

Dear reviewer, we are pleased and appreciate your vigilant and thorough reading of this manuscript and we would like to say thank you for your thoughtful and constructive comments and suggestions. Based on your comments and suggestions, we had carefully corrected the manuscript one by one. And the whole document had revised.

Reviewer #1: The study is very important for the global trachoma community for decision-making at global/international, national, regional, district, and community levels by all stakeholders involved as Ethiopia currently has an estimated 50% of the global population requiring interventions for trachoma elimination. Addressing the trachoma problem in Ethiopia is potentially wiping out half the global trachoma problem. There is enough information that shows that the study is important and worthy of publication. However, major revision is needed. The overall presentation is poor. Grammar is poor. I suggest the authors find someone who can proofread the many grammatic mistakes.

**Author Response:** thank you very much, dear reviewer, a critical revision has been made to the presentation of the revised manuscript, please see the revised version

1. Objectives should be revised. See my comments in the attached file. They are not clearly articulated.

**Author Response**: thank you, dear reviewer, we have notably improved the objective part of the manuscript .please see the revised version

I believe that the study design is appropriate to address the stated objectives.

The population is clearly described and appropriate for the hypothesis being tested.

The sample size looks sufficient to ensure adequate power to address the hypothesis being tested.

I believe that correct statistical analysis was used to support conclusions although I may not be strong enough on statistical methods.

2. Selection of participants is generally ethical and meets regulatory requirements. However, some women were excluded with weak justification for the exclusion. Better explanation needed in the limitation section.

**Author Response:** thank you, dear reviewer, we have included the explanation in the limitation part of the revised manuscript. , Due to the lack of sign language-trained data collectors and the inability to obtain psychiatric therapists as data collectors, it was not possible to include those mothers with mental illness, hearing problems and a mother having a severe systemic illness prevents her from answering our question but their number was quite insignificant. It was only 4 mothers excluded from the study based on our exclusion criteria.

3. Reviewer #1: The study is a replication of similar studies conducted in other parts of the country and in other countries. The analysis matches the analysis plan. Results are completely presented. The authors need to improve the presentation in general

**Author Response:** thank you, dear reviewer, although there are similar studies conducted, this study explored neglected tropical diseases that became hyper-endemic in our study area after the implementation of SAFE for about 8 to 11 years. Other than the three similar settings, in which SAFE was equally implemented, only our study area remained hyper-endemic having a prevalence of 37 %, it remained a mystery. so we were very interested to study the prevention practice of the community and thus the elimination of an estimated half the global burden moreover, this study uses multi-level modeling taking into account the clustering effect to draw valid inferences and conclusions. Hence this study would be important for employing combined efforts to identified modifiable risk factors and will have significant policy implications in providing support to the affected community.

Dear reviewer, we have improved the presentation critically. please see the revised version

4. Reviewer #1: Conclusions are supported by the data presented but presentation is generally poor.

The limitations of analysis are described but more work needed.

The authors tried to discuss how these data can be helpful to advance our understanding of the topic under study, but more work is needed to improve the presentation of the study.

Authors have poorly addressed the public health relevance of the study. I have made some suggestions in the attached file.

**Author Response:** thank you, dear reviewer, we have critically improved the conclusion, and limitation and addressed the public health relevance of the study in the strength part of the study in the revised version.

5. Reviewer #1: The study is a replication of studies conducted in other countries and in other parts of Ethiopia to assess community practices that influence trachoma prevention and elimination. Half the global population requiring intervention for trachoma elimination is in Ethiopia where some regions/districts have up to 37% TF rate (hyperendemicity) after years of A treatment. This study demonstrates the need to consider support for the introduction of other interventions (F and E) for trachoma elimination in Ethiopia and thus elimination of an estimated half the global burden. I believe that the essential information needed (objectives, methodology, result) is available in this draft of the manuscript. However, overall presentation is poor with too many grammatic errors. Authors should take time to improve the presentation including finding someone who can proofread and edit the manuscript for them. I have made some suggestions in the attached file.

**Author Response:** thanks a lot dear reviewer, we have critically improved the newly revised manuscript as a whole one by one please look the revised manuscript.
