# Supplementary material for: Trachoma prevention practice and associated factors among mothers having children aged under nine years in Andabet district, northwest Ethiopia, 2022: A multi-level analysis
Source: PLoS Negl Trop Dis. 2023 Jun 30;17(6):e0011433. doi: 10.1371/journal.pntd.0011433 (PMC10313038; doi:10.1371/journal.pntd.0011433)
Supplement: Attachment — Submitted filename: response for reviewer comment.docx [file pntd.0011433.s005.docx]

**Authors’ response to Editor’s and Reviewer’s comments**

**The title “Trachoma prevention practice and Associated Factors among mothers having children aged under nine Years in Andabet District, northwest Ethiopia, 2022:a multilevel analysis”**

Dear Editor, we have revised our manuscript based on the reviewer's suggestions and comments.  Here is a response to the reviewer's and your comments and suggestions

**Author's response to the editor’s suggestions/comments**

Thank you very much for submitting your manuscript "Trachoma prevention practice and associated factors among mothers having children aged under nine years in Andabet district, northwest Ethiopia, 2022: A multi-level analysis" for consideration at PLOS Neglected Tropical Diseases. As with all papers reviewed by the journal, your manuscript was reviewed by members of the editorial board and by several independent reviewers. The reviewers appreciated the attention to an important topic. Based on the reviews, we are likely to accept this manuscript for publication, providing that you modify the manuscript according to the review recommendations.

**Author Response:** thank you very much, dear editor, for your great notion regarding the manuscript, we modified the manuscript according to the reviewer’s recommendations.

When you are ready to resubmit, please upload the following:

[1] A letter containing a detailed list of your responses to the review comments and a description of the changes you have made in the manuscript. Please note while forming your response, if your article is accepted, you may have the opportunity to make the peer review history publicly available. The record will include editor decision letters (with reviews) and your responses to reviewer comments. If eligible, we will contact you to opt-in or out.

**Author Response:** thank you, dear editor, we have prepared both the letter containing a detailed list of our responses to the review comments and a description of the changes we have made in the manuscript.

[2] Two versions of the revised manuscript: one with either highlights or tracked changes denoting where the text has been changed; the other a clean version (uploaded as the manuscript file).

**Author Response:** we have prepared the two versions of the manuscript one with tracked change and the other a clean version

Figure Files:

While revising your submission, please upload your figure files to the Preflight Analysis and Conversion Engine (PACE) digital diagnostic tool, https://pacev2.apexcovantage.com. PACE helps ensure that figures meet PLOS requirements. To use PACE, you must first register as a user. Then, login and navigate to the UPLOAD tab, where you will find detailed instructions on how to use the tool. If you encounter any issues or have any questions when using PACE, please email us at [figures@plos.org](mailto:figures@plos.org).

**Author Response:** thank you, dear editor, we have uploaded the figure with PACE

Data Requirements:

Please note that, as a condition of publication, PLOS' data policy requires that you make available all data used to draw the conclusions outlined in your manuscript. Data must be deposited in an appropriate repository, included within the body of the manuscript, or uploaded as supporting information. This includes all numerical values that were used to generate graphs, histograms etc.. For an example see here: <http://www.plosbiology.org/article/info%3Adoi%2F10.1371%2Fjournal.pbio.1001908#s5>.

**Author Response:** thank you, dear editor**,** the data used for this study was included with the submission as a supporting information-compressed zip file.

References
please review your reference list to ensure that it is complete and correct. If you have cited papers that have been retracted, please include the rationale for doing so in the manuscript text, or remove these references and replace them with relevant current references. Any changes to the reference list should be mentioned in the rebuttal letter that accompanies your revised manuscript. If you need to cite a retracted article, indicate the article's retracted status in the References list and also include a citation and full reference for the retraction notice.

**Author Response:** thank you very much, dear editor, for reminding us to review the reference list. We have not cited any papers that have been retracted. We only removed the duplicated references and update the citation.

**Response to the reviewer's comment**

Dear reviewer, we are pleased and appreciate your vigilant and thorough reading of this manuscript and we would like to say thank you for your thoughtful and constructive comments and suggestions. Based on your comments and suggestions, we had carefully corrected the manuscript one by one.

1. **Method**: reviewer #1: Manuscript was revised well needing only minor revisions before publication

**Author Response:** thank you very much, dear reviewer, we have carefully done the revision based on your comments and suggestions

2. **Result**: reviewer #1: Manuscript was revised well needing only minor revisions before publication.

**Author Response**: thank you very much, dear reviewer, we have carefully done the revision based on your comments and suggestions

3. **Conclusion**: Reviewer #1: Manuscript was revised well needing only minor revisions before publication

**Author Response:** thank you very much, dear reviewer, we have carefully done the revision based on your comments and suggestions

4. Reviewer #1: Most of the revision needed is on use of acronym. In line 28 please write "trachoma prevention practices" in full followed by the acronym in bracket (TPP). Afterwards, just put TPP. Use the acronym TPP instead of in full in line 31, 47,49, 86-87, 91,92,93, 94,96,118, 151, 160-161, 200,205,210,211-212, 218, 219-220, 231, 252,253, 264-265, 267, 268-269, 269, 271, 281, 283, 283, 287, 289, 291-292, 294, 301, 303,304, 321-322, 323, 329, 335, 336-337, 359, 362, 369. I might have missed some. Please go through and change accordingly.

**Author Response:** thank you very much, dear reviewer, we have carefully gone through and changed based on your comments.

5. Reviewer #1: Abstract: World Health Organization (WHO). I suggest you use 'cleanliness' throughout when referring to the SAFE strategy. Put TPP in bracket after trachoma prevention practices as you have used the acronym later in the abstract. On dates, I believe you say from June 5 to June 10, 2022. Remove 'from' and the date is still good (was conducted June 1-30, 2022

**Author Response:** thank you very much, dear reviewer, we have carefully gone through and changed based on your comments and suggestions.

5. Reviewer #1: Line 25: World Health Organization (WHO).
Line 34: remove 'from'
Line 37: small 'v'
Line 57: for consistency, please use 'cleanliness'.
Line 74: remove the’s’ from disease, use singular as it is just trachoma.
Line 84-85: please improve on this sentence as it is vague, not clear enough what you are saying.
Line 87: small’s’.
Line 100 and 107: remove 'from'.
Line 119: no brackets please.
Line 120: add comma after 'rate'.
Line 127: please replace 'of' with 'that'.
Line 129: small 'c'.
Line 143: please replace highlighted words with 'were' and add a comma after 'visits'.
Line 155: comma after status.
Line 164: add a full stop after the bracket.
Line 167: 'ies' in the bracket, not just’s’ as plural of fly is flies.
Line 174: small’d’.
Line 189: dived or divided?
Line 195: use comma, then small 'w'.
Line 235: capital 'M'.
Line 246: full stop after bracket.
Line 286: Capital 'O'.
Line 306: capital 'S'.
Line 307: i suggest you use 'as in' or because in'.
Line 308: comma after 'besides'.
Line 309: I suggest you use 'smaller'.
Line 309: better to use 'might be'.
Line 310: 'difference' better.
Line 315: use comma, not full stop.
Line 322: better to use 'receive".
Line 328: comma after moreover.
Line 331: space, then capital 'I'.
Line 337: suggest you use 'because'.
Line 338: full stop after minutes.
Line 345: 'a neglected tropical disease', not diseases as you are referring to just trachoma.
Line 346: full stop please.
Line 347: in place of 'to', use 'to address'.
Line 348: Full stop after 'community'. Then start next sentence with a capital B.
Line 351: full stop instead of comma.
Line 352: small’d’.
Line 354-355: sounds negative as written here. I suggest instead "although we do not expect a significant number of such women in the targeted communities".
Line 362-363-364: please rewrite. I suggest “Therefore, special attention should be given to these high-risk groups so that this devastating health problem can be decreased".
Line 366: cleanliness.
Line 367: I suggest 'the WHO-recommended SAFE strategy for elimination of trachoma especially in highly endemic countries like Ethiopia".
Line 371: use 'reside far".
Line 373-374: I suggest "will negatively affect efforts of the ".
Line 374: elimination, not illumination.

**Author Response:** Thank you very much, dear reviewer, for your deep insight, substantial suggestions, and comments. We have carefully done the revision based on your comments and suggestions.

Reviewer #1: I believe that this is a good study conducted in Ethiopia and that this manuscript will again highlight the need for special support for the implementation of the F and E components of the WHO-recommended SAFE strategy in countries like Ethiopia that currently has about 50% of the global trachoma burden if the disease is to be eliminated as a public health problem globally. The authors have significantly improved the presentation.

**Author Response:** thank you very much, dear reviewer, for the great notion you have regarding the study.
